# Reduced neural progenitor cell count and cortical neurogenesis in guinea pigs congenitally infected with *Toxoplasma gondii*

**DOI:** 10.1038/s42003-023-05576-6

**Published:** 2023-11-27

**Authors:** Thomas Grochow, Britta Beck, Zaida Rentería-Solís, Gereon Schares, Pavlo Maksimov, Christina Strube, Lisa Raqué, Johannes Kacza, Arwid Daugschies, Simone A. Fietz

**Affiliations:** 1 Institute of Veterinary Anatomy, Histology and Embryology, Faculty of Veterinary Medicine, Leipzig University, Leipzig, Germany; 2 Institute of Parasitology, Faculty of Veterinary Medicine, Leipzig University, Leipzig, Germany; 3 National Reference Laboratory for Toxoplasmosis, Institute of Epidemiology, Friedrich-Loeffler-Institut, Federal Research Institute for Animal Health, Greifswald-Insel Riems, Germany; 4 Institute for Parasitology, Centre for Infection Medicine, University of Veterinary Medicine Hannover, Hannover, Germany; 5 Veterinary practice Raqué, Leipzig, Germany; 6 BioImaging Core Facility, Faculty of Veterinary Medicine, Leipzig University, Leipzig, Germany

**Keywords:** Central nervous system infections, Infection, Disease model, Developmental disorders

## Abstract

*Toxoplasma (T.) gondii* is an obligate intracellular parasite with a worldwide distribution. Congenital infection can lead to severe pathological alterations in the brain. To examine the effects of toxoplasmosis in the fetal brain, pregnant guinea pigs are infected with *T. gondii* oocysts on gestation day 23 and dissected 10, 17 and 25 days afterwards. We show the neocortex to represent a target region of *T. gondii* and the parasite to infect neural progenitor cells (NPCs), neurons and astrocytes in the fetal brain. Importantly, we observe a significant reduction in neuron number at end-neurogenesis and find a marked reduction in NPC count, indicating that impaired neurogenesis underlies the neuronal decrease in infected fetuses. Moreover, we observe focal microglioses to be associated with *T. gondii* in the fetal brain. Our findings expand the understanding of the pathophysiology of congenital toxoplasmosis, especially contributing to the development of cortical malformations.

## Abstract (web summary)

An in vivo study in guinea pigs suggests that *Toxoplasma gondii* infects neurons, astroglia and neural stem cells (NPCs) in the fetal brain after its diaplacentar transmission leading to reduced numbers of NPCs and neurons, and microglia activation.

## Introduction

The obligate intracellular parasite *Toxoplasma (T.) gondii* causes the zoonosis toxoplasmosis^1–3^. In humans, the worldwide seroprevalence of *T. gondii* is approximately 33%^4,5^. Around 1.9% of women of childbearing age become infected for the first time during pregnancy^5^. All warm-blooded animals can serve as intermediate hosts and members of the cat family (Felidae) are the definitive hosts of *T. gondii*. The life cycle of *T. gondii* consists of different developmental stages^6,7^. This parasite reproduces sexually in the intestinal epithelium of the definitive host, which results in the excretion of oocysts in the feces^8^ In the external environment, during a process called sporulation, infective sporozoites develop within the oocysts. Oral infection of the intermediate host leads to rapid asexual proliferation of the acute (tachyzoite) stage^7,9^. After infection, *T. gondii* tachyzoites disseminates through the infected host via blood, lymph or other body fluids, able to reach all host tissues. About 10–14 days after infection, the immune response of the host against *T. gondii* has developed and causes chronic infection, which is characterized by intracellular tissue cysts, harboring bradyzoites, which may remain in the host for life, especially in the brain, retina, skeletal, and cardiac muscle cells^7,10–13^. Primary routes of infection of the intermediate host are insufficiently heated or raw meat containing tissue cysts and ingestion of water or food contaminated with sporulated oocysts^14–16^.

Importantly, the parasite is able to cross the placental barrier during maternal parasitemia and subsequently infect the embryo/fetus after primary infection of the mother^1,3,17^. In healthy and immunocompetent adults, the infection is usually asymptomatic. However, congenital infection may lead to severe symptoms in the offspring, especially in the brain^6,18^. The clinical picture of human congenital toxoplasmosis depends on the time of infection during pregnancy. An infection in early gestation, especially in the first trimester may lead to abortion. Infection in mid gestation is often characterized by microcephaly, intellectual disability, and retinochorioiditis, while congenital infection in the third trimester of pregnancy may result in cerebral calcifications and hepatosplenomegaly^19,20^.

Data from in vitro experiments and adult mouse models have shown that *T. gondii* is able to infect neurons and astrocytes, leading to impairments in the number, morphology and function of the infected cells^21–28^. In eutherian mammals, neurogenesis occurs predominantly before birth and therefore neural stem and progenitor cells (NPCs) are exclusively present in high abundance in the prenatal cortex. Based on location of their nucleus at mitosis, NPCs can be divided into two major classes: apical progenitors (APs) and basal progenitors (BPs)^29–34^. APs are the primary NPCs, whose cell body resides in the ventricular zone (VZ), the germinal zone adjacent to the lateral ventricle and characteristically express the nuclear marker protein Pax6^35,36^. They first expand by proliferative divisions^37^. With the onset of neurogenesis, APs start dividing asymmetrically, thereby generating BPs that accumulate in the subventricular zone (SVZ). The SVZ is the germinal zone basal to the VZ and constitutes the major site of neuron production in the developing dorsal telencephalon^38–42^. BPs consist of two major subtypes: basal intermediate progenitors and basal radial glia, with the latter sharing key features with APs including the expression of Pax6^41,43–45^ and basal intermediate progenitors characteristically expressing the nuclear marker protein Tbr2^46,47^. Newborn neurons migrate radially into the cortical plate (CP) in a birth date-dependent *inside*-*out* manner, in which later-born neurons migrate and settle past earlier-born neurons^48–50^.

Up to date, the host cells and alterations of *T. gondii* infection in the brain of human fetuses congenitally infected with *T. gondii* are still poorly understood, due to the difficulties in performing clinical studies in pregnant women. As such, whether *T. gondii* infects NPCs in the fetal brain and what the potential consequences on brain development are, remains currently unknown. The aim of this study is to determine the in vivo host cells of *T. gondii* and the developmental alterations associated with a congenital *T. gondii* infection in the fetal brain, specifically focusing on the neocortex. The study was conducted in the guinea pig, which represents a highly suitable animal model for human congenital toxoplasmosis, due to the similarities in brain maturity status at birth, in relative timing of cortical neurogenesis and in placentation to humans^51–53^. Here, we demonstrate that *T. gondii* infects NPCs, neurons and astrocytes in the fetal guinea pig brain following vertical transmission of the parasite. Our data show that *T. gondii* infection of the dam during first trimester of gestation results in massive changes in neocortex development of the fetus. Specifically, we observed a significant reduction in the number of neurons at end-neurogenesis and found a marked reduction in the number of BPs, specifically Pax6 and Tbr2-expressing BPs, in the fetal neocortex during mid-neurogenesis, indicating that impaired neurogenesis underlies the neuronal decrease in *T. gondii* infected fetuses. Moreover, we found *T. gondii* tachyzoites to be associated with microglia nodes in the fetal brain as early as second trimester of gestation.

## Results

### Fetuses were successfully infected with *T. gondii* 17 and 25 days after inoculation of dams

All guinea pig dams became pregnant upon first mating. No clinical abnormalities, i.e., in activity, movement, hair coat, water and food intake, breathing, mucosal color, urine, feces were detected in any of the dams during the daily assessment (Supplementary Table 1). Moreover, no abnormalities were found in the pregnant animals during the ultrasonographic and gynecological examination and no resorptions, abortions or stillbirth of fetuses were observed during the entire experiment. Litter size (mean ± SEM) of dams was 2.33 ± 0.29 for the control and 3.44 ± 0.34 for the infection groups.

Prior to infection, all blood samples of the control and infection groups were seronegative using immunoblotting against a *T. gondii* p30 surface antigen. In order to verify a *T. gondii* infection of the dam and fetus, tissue samples were obtained from distinct organs, i.e., maternal heart, liver, and spleen and fetal brain, heart, liver, spleen, thigh muscle, placenta and amniotic fluid, and analyzed for the presence of *T. gondii* DNA by qPCR targeting a *T. gondii* specific 529 base pair repeat element. In all dams and fetuses of the control groups, no *T. gondii* DNA was detected in any of the maternal and fetal tissues and organs analyzed (Supplementary Table 2, lines 43–74). In all dams of the infection groups, *T. gondii* DNA was detected in at least one maternal organ, indicating that all dams were successfully infected (Supplementary Table 2, lines 1, 6, 11, 14, 17, 22, 27, 32, 36). In all fetuses of dams dissected on gestation day 33, i.e., 10 days after inoculation of dams, no *T. gondii* DNA was detected in any of the tissues and organs analyzed (Supplementary Table 2, lines 2–5, 7–10, 12, 13). Thus, infection of fetuses dissected on gestation day 33 was considered as unsuccessful and the corresponding fetuses were excluded from further analysis. In all fetuses of dams dissected on gestation days 40 and 48, i.e., 17 and 25 days after inoculation of dams, *T. gondii* DNA was detected in at least two tissues and organs of each fetus, indicating their successful infection (Supplementary Table 2, lines 15, 16, 18–21, 23–25, 28–31, 33–35, 37–41).

### *T. gondii* DNA was present in various fetal tissues and organs including the brain

Using qPCR analysis, *T. gondii* DNA was detected in all fetal placentas and in the majority of the fetal brain (89%), heart (78%) and spleen (67%) samples on gestation day 40 (Supplementary Table 2, line 26). By gestation day 48, the percentage of fetal tissues and organs containing *T. gondii* increased with its DNA being present in all fetal placental, amniotic fluid, brain and heart samples and in the majority of the fetal spleen (58%) and thigh muscle (78%) samples (Supplementary Table 2, line 42).

**Fig. 1:**
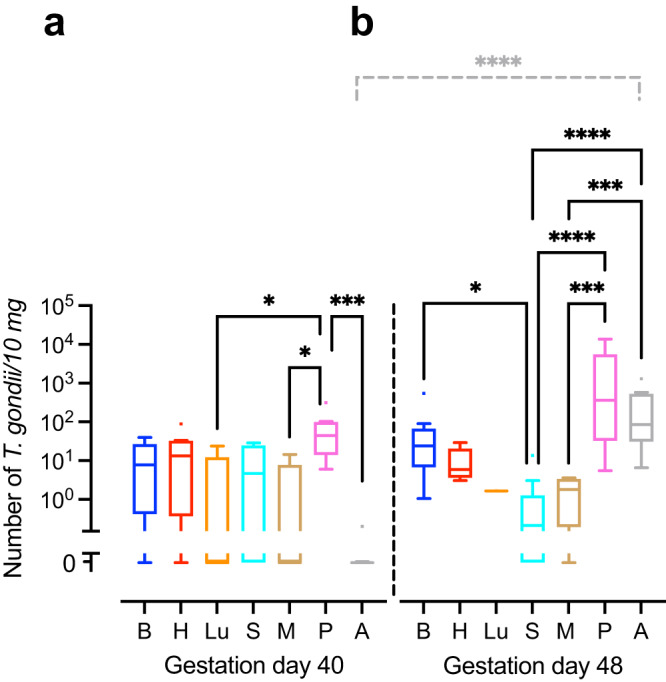
*T. gondii* DNA loads in the guinea pig fetus congenitally infected with *T. gondii*. **a**, **b** *T. gondii* DNA loads in 10 mg tissue were obtained by qPCR in fetuses of dams inoculated with *T. gondii* on gestation day 23 and dissected on day 40 (**a**) and 48 (**b**). Center line, median; box limits, upper and lower quartiles; whiskers, 1.5× interquartile range; points, outliers. B brain, H heart, Lu lung, S spleen, M thigh muscle, P fetal placenta, A amniotic fluid. Solid lines indicate comparison of fetal tissues and organs obtained from dams infected for the same duration of infection. Dashed lines indicate comparison between the same tissue and organ within the different durations of infection. *P < 0.05; ***P < 0.001; ****P < 0.0001. Individual *P* values: comparison between tissues and organs on gestation day 40: *P* = 0.0433, P vs Lu; *P* = 0.0108, P vs M; *P* = 0.0002, P vs A; comparison between tissues and organs on gestation day 48: *P* = 0.0172 B vs S; *P* < 0.0001, P vs S, *P* < 0.0001, A vs S; *P* = 0.0001, P vs M; *P* = 0.0005, A vs M; comparison between gestation days 40 and 48: *P* < 0.0001, A. Only statistically significant differences are shown. Individual n values represent biologically independent samples: comparison between tissues and organs on gestation day 40: *n* = 9, B; *n* = 9, H; *n* = 5, Lu; *n* = 9, S; *n* = 9, M; *n* = 8, P; *n* = 9, A; comparison between tissues and organs on gestation day 48: *n* = 12, B; *n* = 11, H; *n* = 1, Lu; *n* = 11, S; *n* = 9, M; *n* = 11, P; *n* = 12, A.

We next translated the Ct values obtained by qPCR into genomic equivalents of tachyzoites by interpolation from a parasite dilution curve and compared values from the same tissue or organ between different durations of infection, i.e., duration between inoculation of the dam and sampling of animals (Fig. 1). No significant differences were found between the *T. gondii* DNA loads of the intra-embryonic tissues and organs, i.e., brain, heart, lung, spleen and thigh muscle when different durations of infection were compared (Fig. 1a, b). However, *T. gondii* DNA load in the amniotic fluid was significantly higher in fetuses dissected on gestation day 48, i.e., 25 days after inoculation of dams, when compared with those infected for a shorter duration, i.e., dissected on gestation day 40, thus 17 days after inoculation of dams (Fig. 1a, b).

When *T. gondii* DNA loads were compared between different tissues and organs within the same group of duration of infection, *T. gondii* DNA loads in fetuses dissected on gestation day 40 were significantly higher in the placenta than in the amniotic fluid and distinct intra-embryonic tissues and organs, i.e., thigh muscle and lung (Fig. 1a). Moreover, in fetuses dissected on gestation day 48, *T. gondii* DNA loads were significantly higher in the placenta and amniotic fluid than in the thigh muscle and spleen (Fig. 1b). No marked differences were found when *T. gondii* DNA loads were compared between the intra-embryonic tissues and organs, i.e., brain, heart, lung, spleen and muscle, on gestation day 40 (Fig. 1a). However, upon longer duration of infection, dissected on gestation day 48, i.e., 25 days after inoculation of dams, highest median *T. gondii* DNA loads in the fetus were observed in the brain being significantly higher when compared with the spleen (Fig. 1b).

### The forebrain constitutes a main target region of *T. gondii* in the fetal brain

**Fig. 2:**
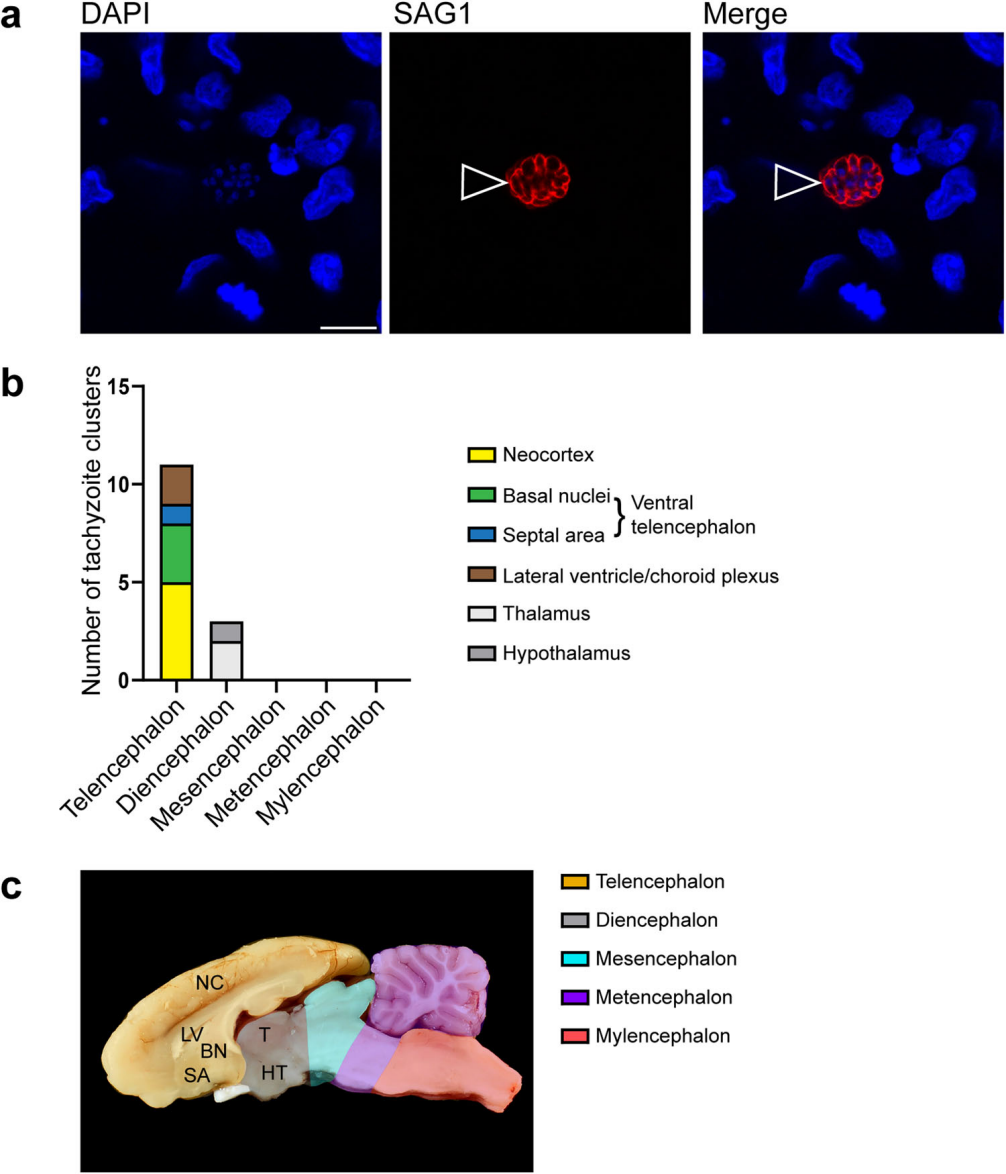
Localization of *T. gondii* tachyzoite clusters in the fetal guinea pig brain. **a** DAPI staining (blue) and immunofluorescence for SAG1 (red) on a 30 µm brain section of a guinea pig fetus of a dam inoculated with *T. gondii* on gestation day 23 and dissected on day 48. Open arrowheads indicate a SAG1+ *T. gondii* tachyzoite cluster. Scale bar, 10 µm. **b** Number of tachyzoite clusters detected by SAG1 immunofluorescence in the distinct brain regions of guinea pig fetuses of dams inoculated with *T. gondii* on gestation day 23 and dissected on days 40 and 48. Data are from all guinea pig fetal brains with Ct value below 35 as determined by qPCR for *T. gondii* (*n* = 5 biologically independent samples for gestation day 40, *n* = 10 biologically independent samples for gestation day 48, Supplementary Table 1). **c** Median section of an adult guinea pig brain. NC neocortex, LV lateral ventricle, BN basal nuclei, SA septal area, T thalamus, HT hypothalamus.

We next investigated, whether there is a specific target region of *T. gondii* within the guinea pig fetal brain. For this, we analyzed 5 randomly chosen sections per brain of *T. gondii* infected fetuses dissected on gestation days 40 and 48 by immunohistochemistry using an antibody for the *T. gondii* tachyzoite*-*specific surface antigen SAG1^54,55^ and quantified delineated SAG1+ clusters (Fig. 2a). A SAG1+ cluster most likely represents a spatially delimited accumulation of *T. gondii* tachyzoites consisting of a primary tachyzoite, which initially invades the host cell, and its daughter cells, which arise from several cycles of endodyogeny^7,56^. We found *T. gondii* tachyzoite clusters to be present in one out of five (20%) brains of fetuses dissected on gestation day 40 and 4 out of 10 (40%) brains dissected on gestation day 48. When different brain regions, i.e., telencephalon, diencephalon, mesencephalon, metencephalon and myeloncephalon, were compared, all *T. gondii* tachyzoite clusters were located in the forebrain, i.e., telen- and diencephalon, with highest numbers being present in the neocortex (Fig. 2b). No *T. gondii* tachyzoite clusters were observed in brain regions caudal to the diencephalon (Fig. 2b). To verify the presence of *T. gondii* tissue cysts, we performed SAG1 immunohistochemistry together Dolichos biflorus agglutinin (DBA) staining on adjacent sections, in which *T. gondii* were detected by SAG1 immunohistochemistry (Supplementary Fig. 1). DBA binds to *N*-acetylgalactosamine moieties present on the *T. gondii* cyst wall^57^. *T. gondii* tissue cysts were solely detected in one section of a fetus dissected on gestation day 48. All cysts contained SAG1+ parasites, i.e., tachyzoites, thus indicating that they are at a very early stage of development.

### *T. gondii* infects neural progenitor cells, neurons, and astrocytes in the fetal forebrain

**Fig. 3:**
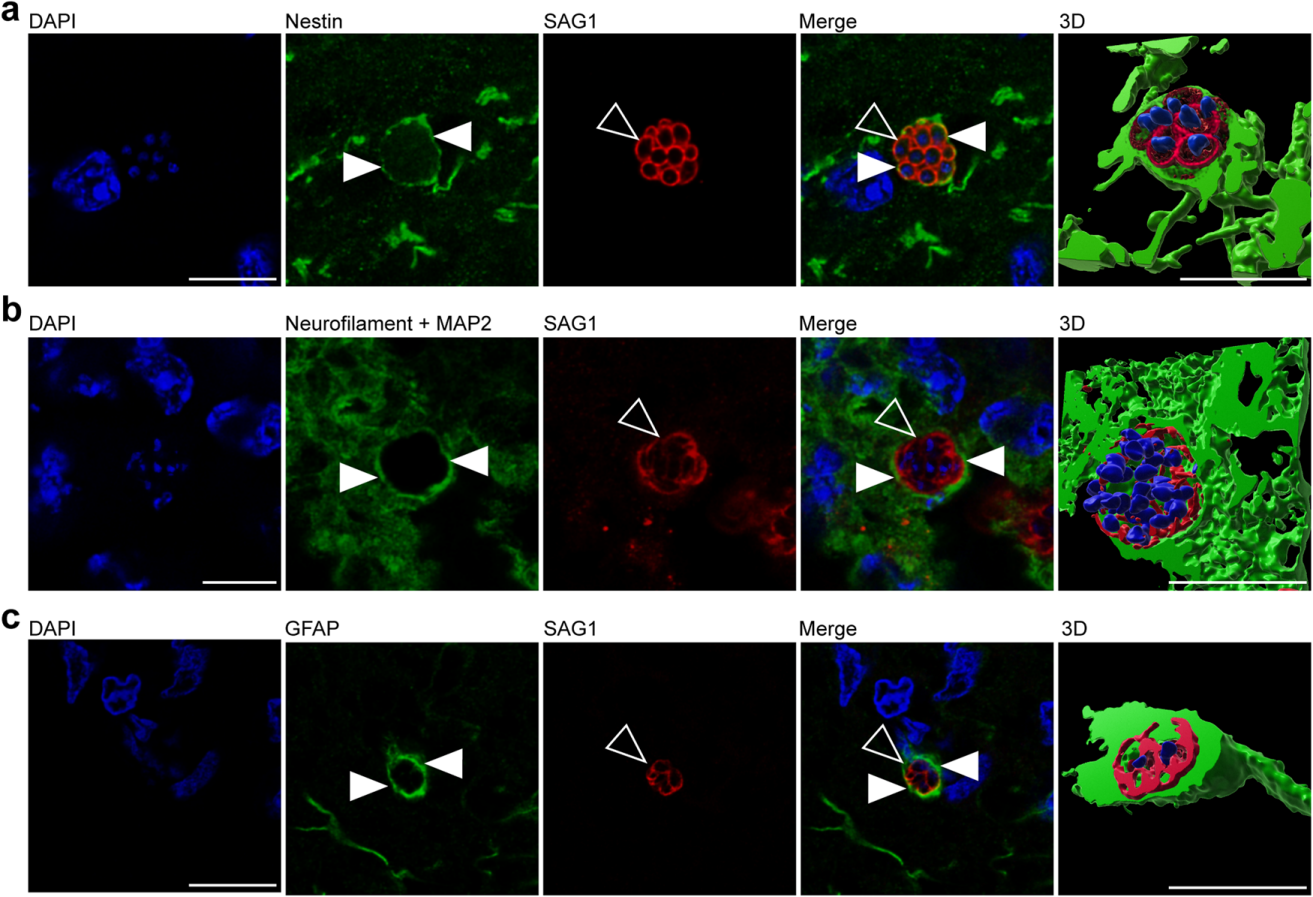
Host cells of *T. gondii* in the fetal guinea pig brain. **a**, **b** DAPI staining (blue) and immunofluorescence for Nestin (green, **a**), neurofilament H and MAP2 (green, **b**), or GFAP (green, **c**), and SAG1 (red) on 30 µm brain cryosections of guinea pig fetuses of dams inoculated with *T. gondii* on gestation day 23 and dissected on day 40 (**b**) or 48 (**a**, **c**). Image showing Nestin immunofluorescence represents a maximum intensity projection of 3 optical sections with a thickness of 0.2 µm each (**a**). Image showing MAP2 immunofluorescence represents a maximum intensity projection of 2 optical sections with a thickness of 0.2 µm each (**b**). Closed arrowheads indicate Nestin+ (**a**), neurofilament H/MAP2+ (**b**), or GFAP+ (**c**) cytoplasm. Open arrowheads indicate SAG1+ tachyzoites. 3D graphics were created by surface rendering for visualization only using Imaris. Scale bars, 10 µm.

In a further step, we elucidated, which specific cell types are infected by *T. gondii* in the guinea pig fetal brain. To this end, we analyzed cortical sections of *T. gondii* infected fetuses dissected on gestation days 40 and 48 by double or triple immunohistochemistry using antibodies for SAG1 and one of the following cytoplasmic marker protein(s): (i) Nestin, an intermediate filament protein of NPCs^58^, (ii) MAP2, a microtubule-associated protein of neuronal dendrites and soma^59^, and neurofilament H, an intermediate filament protein of neuronal axons^60,61^, and (iii) GFAP, an intermediate filament protein of astrocytes^62,63^. Interestingly, *T. gondii* tachyzoites were completely encapsulated by Nestin+ immunofluorescence signal (Fig. 3a), thus revealing their presence in NPCs. Moreover, *T. gondii* tachyzoites were detected in neurons and astrocytes as visualized by the complete encapsulation of MAP2 and neurofilament H (Fig. 3b), and GFAP immunofluorescence signal, respectively (Fig. 3c). Together, this demonstrates the ability of *T. gondii* to infect NPCs, neurons as well as astrocytes in the fetal guinea pig brain following its vertical transmission.

### *T. gondii* infection results in a decrease of neurons and NPC count in the fetal neocortex

**Fig. 4:**
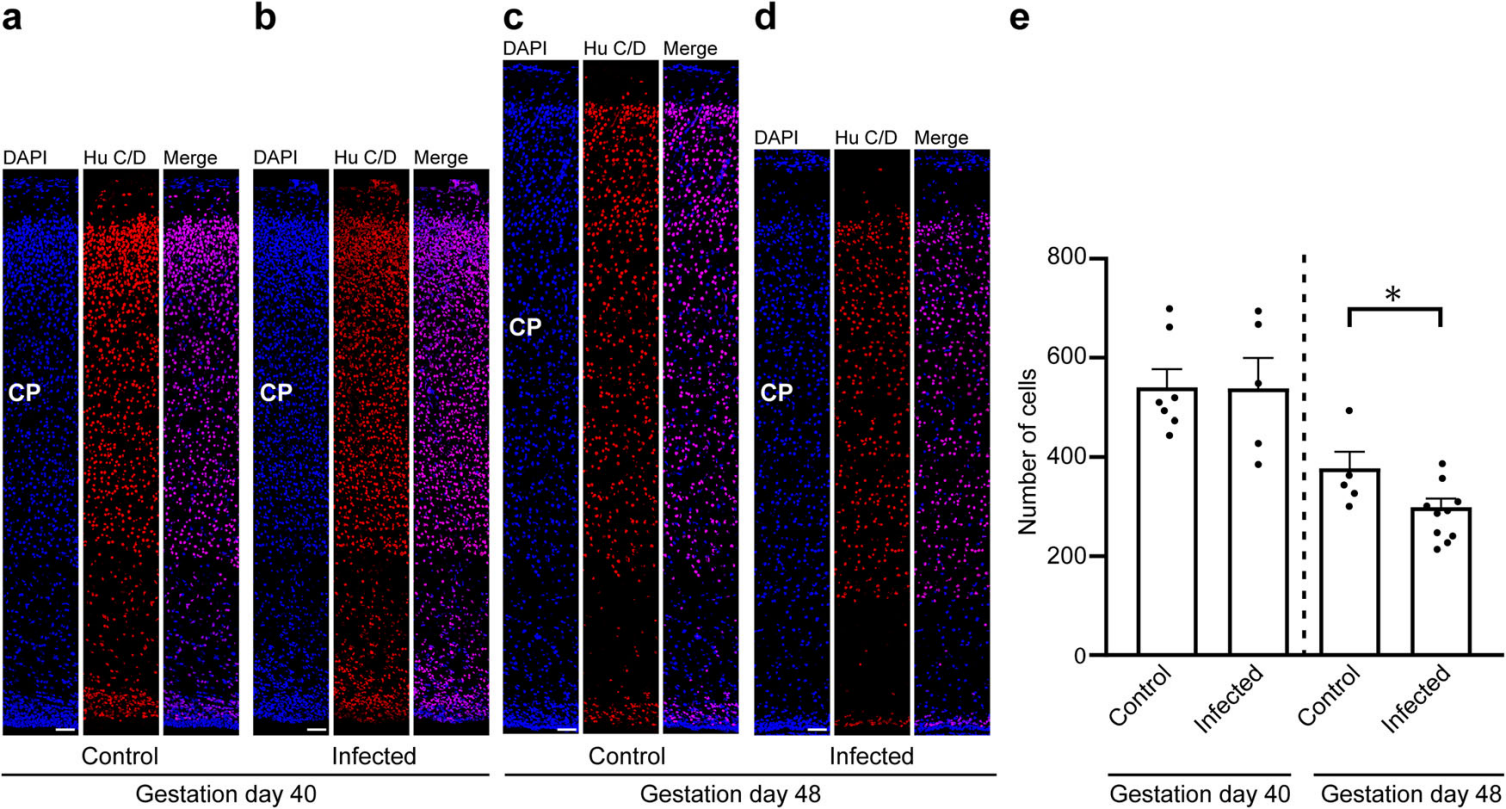
Hu C/D expression in the fetal guinea pig neocortex. **a**–**d** DAPI staining (blue) and immunofluorescence labeling for Hu C/D (red) on 30 µm brain cryosections of guinea pig fetuses of control dams (**a**, **c**) or dams inoculated with *T. gondii* on gestation day 23 (**b**, **d**) and dissected on day 40 (**a**, **b**) or 48 (**c**, **d**). The entire cortical wall is shown. CP, cortical plate. Scale bars, 50 µm. **e** Quantification of Hu C/D+ cells in the cortical wall, expressed as number of cells per 100 µm ventricular surface. Cortical wall corresponding to a total ventricular surface of 150–800 µm was analyzed. Center line, mean; error bar, SEM. Data of the infection groups are from all guinea pig brains with Ct value below 35 as determined by qPCR for *T. gondii* (*n* = 5 for gestation day 40, *n* = 10 biologically independent samples for gestation day 48, Supplementary Table 1). Data of the control group are as follows: *n* = 7 biologically independent samples for gestation day 40, *n* = 5 biologically independent samples for gestation day 48. *P < 0.05 (*P* = 0.0181, df = 13).

To gain insight into the direct implications of the *T. gondii* infection in the guinea pig fetal neocortex, specifically on the pool size of the infected cells, we analyzed cortical sections at a medium position of the lateral ventricle per brain of the control and infection groups by immunohistochemistry using antibodies for characteristic marker proteins of the cells of interest. We first focused on neurons and used an antibody for the pan-neuronal marker Hu C/D (Fig. 4a–d)^64,65^. Quantification of Hu C/D+ cells revealed no significant differences in neuron number between fetuses of the control and the infection group dissected on gestation day 40 (Fig. 4a, b, e). However, on gestation day 48, the number of Hu C/D+ cells was significantly lower in the neocortex of fetuses infected with *T. gondii* when compared with those of the control group (Fig. 4c–e). No major differences in the distribution of Hu C/D+ cells between the control and infection groups on both gestation days were obvious, indicating that the apical-basal migration of neurons is not affected in the *T. gondii* infected brains.

**Fig. 5:**
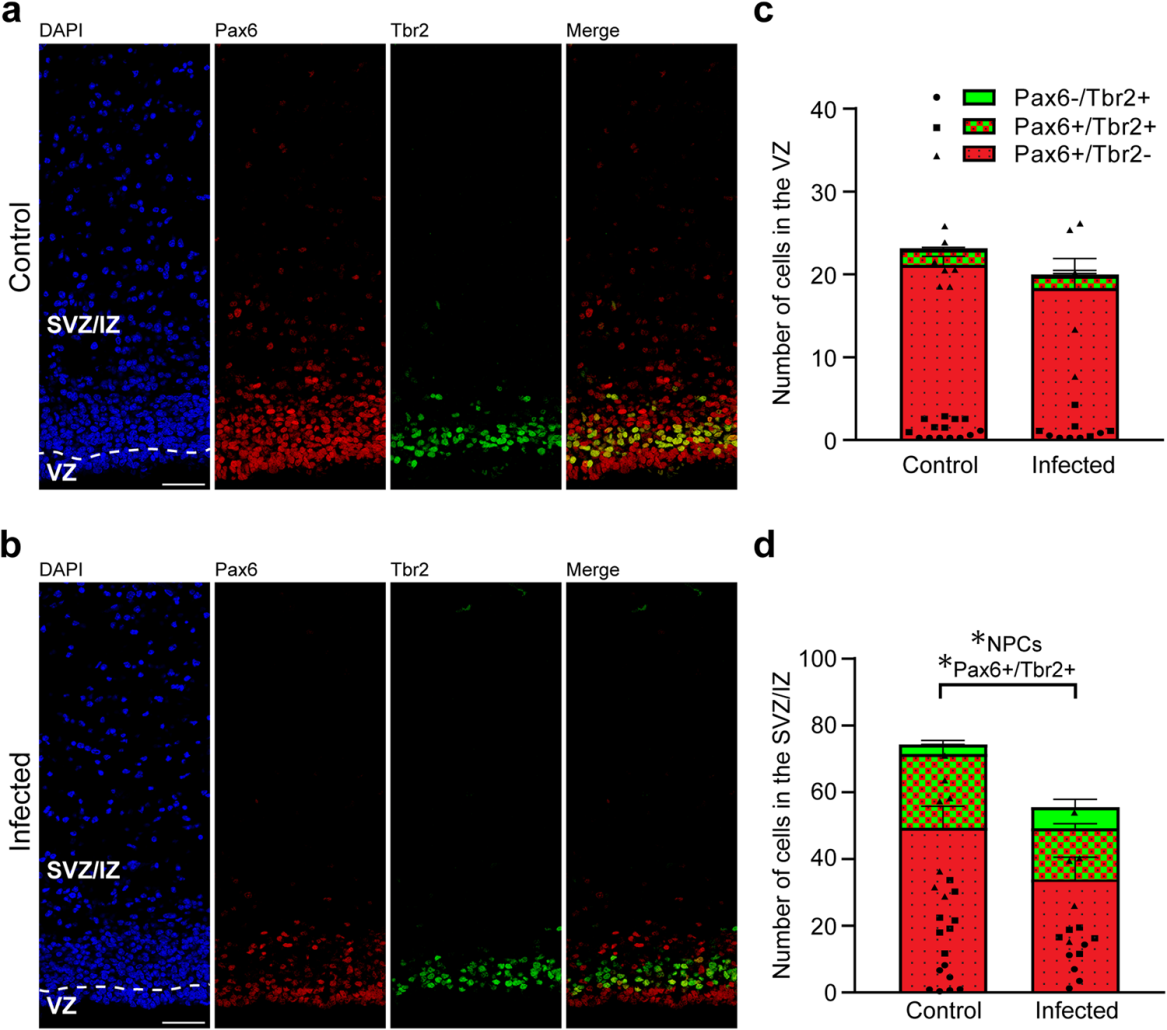
Pax6 and Tbr2 expression in the fetal guinea pig neocortex. **a**, **b** DAPI staining (blue) and immunofluorescence labeling for Pax6 (red) and Tbr2 (green) on 30 µm brain cryosections of guinea pig fetuses of control dams (**a**) or dams inoculated with *T. gondii* on gestation day 23 (**b**) and dissected on day 40. Scale bar, 50 µm. **c**, **d** Quantification of Pax6+ (red) and/or Tbr2+ (green) NPCs in the ventricular zone (VZ, **c**) and subventricular zone/intermediate zone (SVZ/IZ), **d**), expressed as number of cells per 100 µm ventricular surface. Cortical wall corresponding to a total ventricular surface of 230–500 µm was analyzed. The dashed line indicates the border between VZ and SVZ/IZ. Center line, mean; error bar, SEM. Data of the infection groups are from all guinea pig brains with Ct value below 35 as determined by qPCR for *T. gondii* (*n* = 5 for gestation day 40, biologically independent samples, Supplementary Table 1). Control group: *n* = 7 biologically independent samples for gestation day 40. *P < 0.05 (*P* = 0.0440, df = 10, NPCs, i.e., the sum of Pax6+/Tbr2–, Pax6+/Tbr2+, Pax6–/Tbr2+ cells, in SVZ/IZ; *P* = 0.0441, df = 10, Pax6+/Tbr2+ cells in SVZ/IZ).

We next analyzed cortical sections by double immunohistochemistry using antibodies for distinct NPC markers, i.e., Pax6, a transcription factor characteristically expressed by APs and basal radial glia, and Tbr2, a transcription factor characteristically expressed by basal intermediate progenitors (Fig. 5a, b)^41,43,44,46^. As shown previously^66^, no Tbr2+ cells were detected in the neocortex of the control and infected groups on gestation day 48, indicating that cortical neurogenesis in the guinea pig has terminated before (Supplementary Fig. 2). On gestation day 40, which constitutes mid-neurogenesis in the guinea pig neocortex^66^, no significant differences in the number of Pax6+ and/or Tbr2+ cells of the VZ between the control and infected fetuses were detected (Fig. 5c), indicating that the pool size of the primary NPCs, i.e., APs, is largely unaffected in the developing neocortex of fetuses infected with *T. gondii* when compared with that of controls. We then focused our analysis on the secondary NPCs in the SVZ/IZ, i.e., BPs. Interestingly, their total number, i.e., the sum of Pax6+/Tbr2–, Pax6+/Tbr2+, Pax6−/Tbr2+ NPCs, on gestation day 40 was significantly lower in *T. gondii* infected fetuses when compared with that of controls (Fig. 5d). When investigating whether this reduction was attributable to a specific BP subpopulation, we found the number of Pax6+/Tbr2+ BPs to be significantly decreased in *T. gondii* infected fetuses when compared with controls (Fig. 5d). Together, our data show that *T. gondii* infection of NPCs and neurons in the fetal guinea pig neocortex impacts their number, resulting in a reduction of the NPC pool during mid-neurogenesis and that of neurons at end-neurogenesis. No significant differences were observed when the cortical thickness was compared between the control and infection group of both gestation days (Supplementary Fig. 3). Together, this suggests that the reduced cell numbers lead to a decrease in cell density but not to a decrease in cortical wall thickness in the brain of *T. gondii* infected fetuses.

**Fig. 6:**
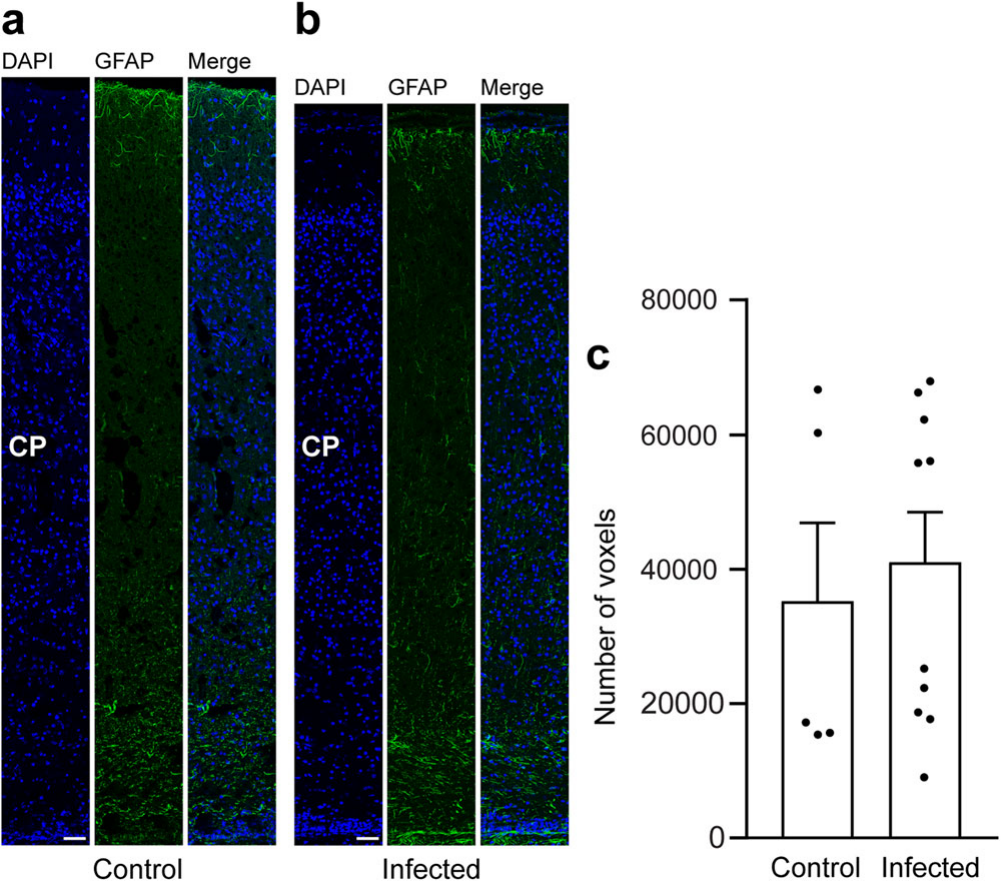
GFAP expression in the fetal guinea pig neocortex. **a**, **b** DAPI staining (blue) and immunofluorescence for GFAP (green) on 30 µm brain cryosections of guinea pig fetuses of control (**a**) or infected (**b**) dams dissected on gestation day 48. Dams were inoculated with *T. gondii* on gestation day 23. Scale bar, 50 µm. The entire cortical wall is shown. CP, cortical plate. Scale bars, 50 µm. **c** Quantification of GFAP+ labeling intensity in the cortical wall, expressed as number of voxels per 100 µm ventricular surface. Cortical wall corresponding to a total ventricular surface of 150–250 µm was analyzed. Center line, mean; error bar, SEM. Data of the infection groups are from all guinea pig brains with Ct value below 35 as determined by qPCR for *T. gondii* (*n* = 10 biologically independent samples, for gestation day 48, Supplementary Table 1). Data of the control group are as follows: *n* = 5 biologically independent samples. (*P* = 0.3325, df = 13).

In a final step, we focused on astrocytes and analyzed cortical sections by immunohistochemistry using an antibody for GFAP (Fig. 6a, b). Similar to previous observations^66^, mature astrocytes, which exhibit a typical star-shaped appearance, were first detected after the end of neurogenesis, i.e., on gestation day 48 in the guinea pig neocortex (Fig. 6a, b). We then quantified the GFAP immunofluorescence intensity in the control and infected fetuses obtained on gestation day 48 and found no difference when the two groups were compared (Fig. 6c). This indicates that *T. gondii* infection of the fetal guinea pig neocortex does not affect the number of astrocytes.

### *T. gondii* infection of the fetal neocortex is not associated with an increase in cell death

Previous studies demonstrated the ability of *T. gondii* to induce cell death^67–69^. In order to investigate whether cell death accounts for the observed NPC and neuron loss in the *T. gondii* infected guinea pig neocortex, we analyzed cortical sections of the control and infection groups by immunohistochemistry using an antibody for caspase-3, a marker of programmed cell death (Supplementary Fig. 4)^70,71^. Only a few apoptotic cells were detected in the cortical wall of the control group at both stages analyzed (Table 1). Similarly, the number of apoptotic cells in the cortical wall of fetuses dissected on gestation days 40 and 48 was markedly low (Table 1). No significant difference in their number was detected when the control and infection groups were compared (Table 1). Moreover, we analyzed DAPI-stained nuclei for pyknosis and/or karyorrhexis, which are hallmarks of cell death, in the cortical wall of the neocortex of both infection groups. We found all pyknotic and fragmented nuclei to be present in caspase-3+ cells. Together, this suggests that cell death is unlikely to account for the reduction in NPC and neuron count observed in the neocortex of *T. gondii* infected fetuses.

**Table 1:** Quantification of apoptotic cells in the cortical wall of fetuses of the control and *T. gondii* infection groups.

Fetus no.	Group	Gestation day	Number of apoptosis^a^	Cortical zone^b^	Mean ± SEM^c^
4A	Infected	40	1	SVZ	0.6 ± 0.4
4B	Infected	40	0		
5A	Infected	40	2	SVZ	
5B	Infected	40	0		
5D	Infected	40	0		
7A	Infected	48	0		0.4 ± 0.1633
7B	Infected	48	1	SVZ	
8A	Infected	48	1	SVZ	
8B	Infected	48	0		
8C	Infected	48	0		
9A	Infected	48	1	CP	
9B	Infected	48	0		
9C	Infected	48	0		
9D	Infected	48	0		
9E	Infected	48	1	CP	
10A	Control	40	0		0.7143 ± 0.4738
10B	Control	40	0		
11A	Control	40	0		
11B	Control	40	0		
11C	Control	40	2	SVZ	
12A	Control	40	0		
12B	Control	40	3	SVZ	
13A	Control	48	0		0
14A	Control	48	0		
14B	Control	48	0		
15A	Control	48	0		
15B	Control	48	0		

*SVZ* subventricular zone, *CP* cortical plate.

^a^Number of apoptotic cells identified by immunohistochemistry for activated caspase 3 and quantified in the cortical wall per 100 µm ventricular surface.

^b^Cortical zone in which apoptotic cells were detected.

^c^Mean ± SEM of the number of apoptotic cells quantified in the entire cortical wall per 100 µm ventricular surface of the infection and control groups. For the infection groups, data are from all guinea pig fetal brains with Ct value below 35 as determined by qPCR for *T. gondii*. Mann–Whitney test was used to compare the number of apoptotic cells quantified in the cortical wall between the control and infection group; *P* (gestation day 40) = 0.5758, *P* (gestation day 48) = 0.1538).

### Focal microglioses are present in the brain of *T. gondii* infected fetuses

**Fig. 7:**
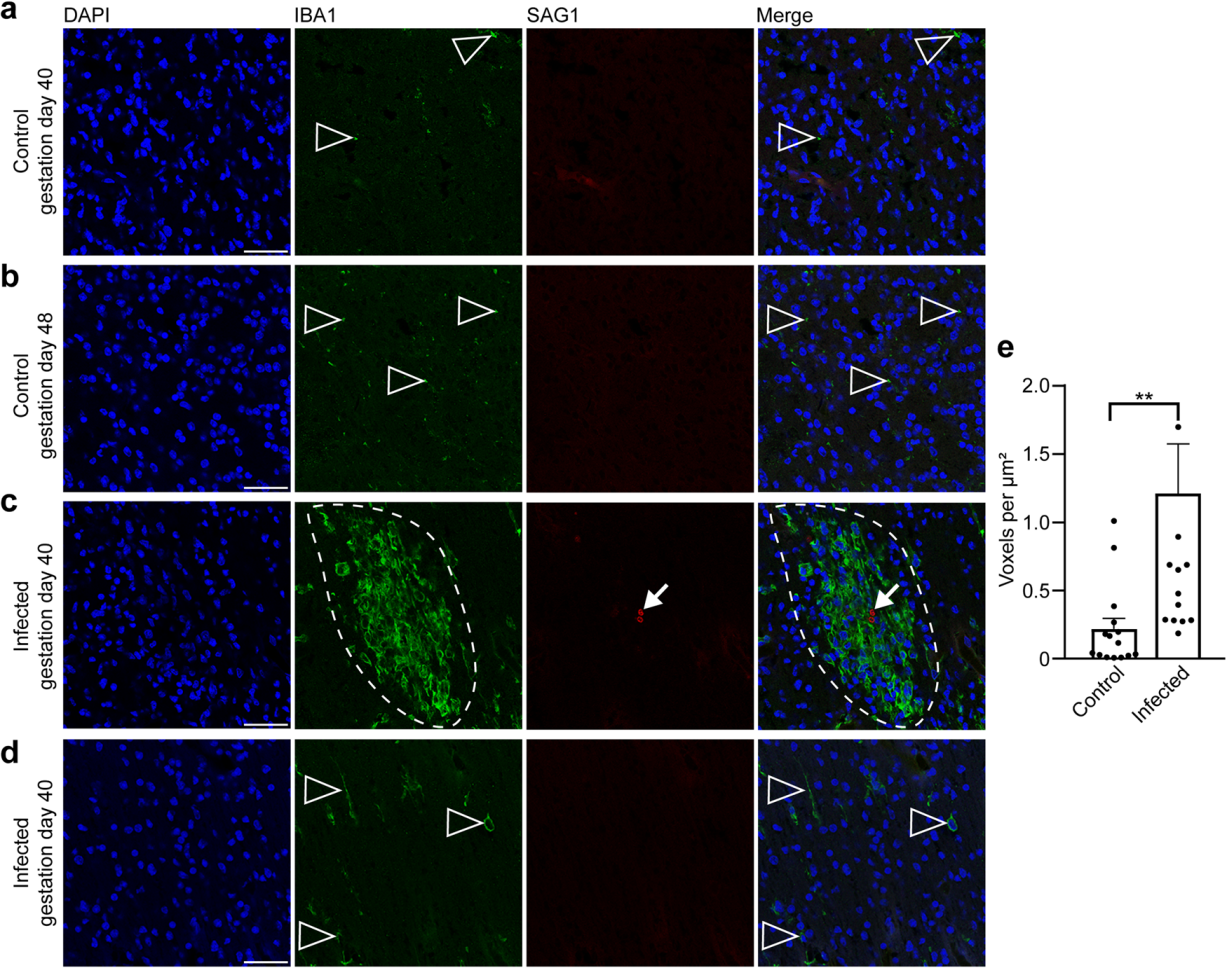
Localization of microglia in foci of *T. gondii* tachyzoite clusters the fetal guinea pig brain. **a**–**c** DAPI staining (blue) and immunofluorescence for Iba1 (green) and SAG1 (red) on 30 µm brain cryosections of guinea pig fetuses of control dams dissected on gestation days 40 (**a**) or 48 (**b**) and dams inoculated with *T. gondii* on gestation day 23 and dissected on day 40 (**c**, **d**). Scale bar, 50 µm. Arrowheads indicate Iba1+ microglia. Arrows indicate SAG1+ *T. gondii* tachyzoites. Dashed line outlines an agglomeration of Iba1+ microglia surrounding SAG1+ *T. gondii* tachyzoites. Image in (**d**) shows the same section and brain region as in (**c**) without the presence of tachyzoites. **e** Quantification of Iba1 labeling intensity as detected by immunofluorescence labeling for Iba1. Labeling intensities were quantified in a 200 × 200 µm area including a tachyzoite cluster of guinea pig fetuses of dams inoculated with *T. gondii* on gestation day 23 and from a 200 × 200 µm area covering the same brain region of control fetuses, dissected on gestation days 40 and 48. Results were expressed as voxels per µm^2^. Center line, mean; error bar, SEM. Data are from 15 *T. gondii* tachyzoite clusters observed in 5 fetuses dissected on gestation days 40 and 48 (Fig. 2) and the corresponding regions of the control groups. **P < 0.01 (*P* = 0.0064, df = 28, *n* = 15 biologically independent samples). Dots not displayed for the infected group: 4.01, 2.52, 4.62.

In the following, we aimed to gain insight into the fetal immune response against *T. gondii* in the guinea pig neocortex, focusing on microglia, which represent the resident immune cells in the central nervous system first^72,73^. For this, the adjacent section of the brain section in which *T. gondii* tachyzoites were detected and a control section of a corresponding brain area of fetuses dissected on gestation days 40 and 48 were stained with antibodies for Iba1, a characteristic marker of microglia^74,75^ and SAG1 (Fig. 7). In fetuses of the control group, microglia were present in the neocortex as early as gestation day 40, i.e., during mid-neurogenesis (Fig. 7a). Remarkably, in the brain of guinea pig fetuses of both infection groups, all *T. gondii* tachyzoites were found in the center of the microglia aggregations and microglia nodules were only present in association with tachyzoites (Fig. 7c, d). Quantification of Iba1 immunofluorescence intensity in the tachyzoite related lesions and corresponding brain areas of the control group revealed a significant increase in the Iba1 fluorescence labeling intensity in the brain of *T. gondii* infected fetuses (Fig. 7e). This suggests that parasitic lesions are associated with focal or multifocal microglioses in the brain of guinea pig fetuses 17 days after inoculation of dams with *T. gondii*.

During activation in response to various stimuli, e.g., brain infection, microglia undergo distinct morphological changes. Non-activated microglia mainly exhibit a ramified morphology, whereas fully activated microglia acquire an amoeboid shape^76^. In order to analyze whether *T. gondii* infection is associated with a change in microglia morphology in the fetal brain, microglia in the tachyzoite related lesions and corresponding brain areas of the control group of fetuses dissected on gestation days 40 and 48 were classified as activated and non-activated using Imaris surface classification (Supplementary Fig. 5). This revealed that tachyzoite related lesions contain a higher proportion of amoeboid to ramified microglia than corresponding control areas. Together, this indicates that a fetal immune response against *T. gondii* associated with the presence of formation of activated microglia nodules is established in the brain of *T. gondii* infected fetuses as early as gestation day 40.

**Fig. 8:**
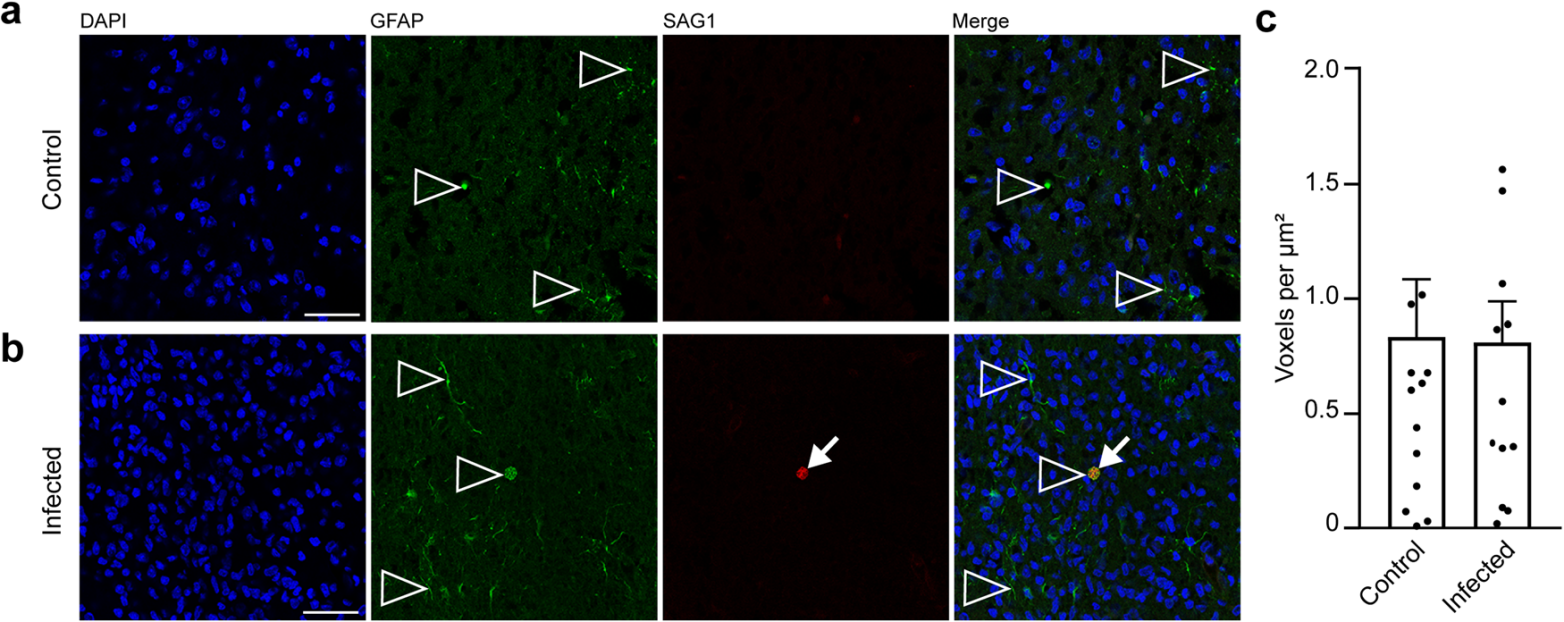
Localization of astroglia in foci of *T. gondii* tachyzoite clusters in the fetal guinea pig brain. **a**, **b** DAPI staining (blue) and immunofluorescence for GFAP (green) and SAG1 (red) on 30 µm brain cryosections of guinea pig fetuses of control (**a**) or infected (**b**) dams dissected on gestation day 48. Dams were inoculated with *T. gondii* on gestation day 23. Scale bar, 50 µm. Arrowheads indicate GFAP+ astroglia. Arrows indicate SAG1+ *T. gondii* tachyzoites. **c** Quantification of GFAP labeling intensity as detected by immunofluorescence labeling for GFAP. Intensities were quantified in a 200 × 200 µm area including a tachyzoite cluster of guinea pig fetuses of dams inoculated with *T. gondii* on gestation day 23 and from a 200 × 200 µm area covering the same brain region of control fetuses, dissected on days 40 and 48. Results were expressed as voxels per µm². Center line, mean; error bar, SEM. Data are from 15 *T. gondii* tachyzoite clusters observed in 5 fetuses dissected on gestation days 40 and 48 (Fig. 2) and the corresponding regions of the control groups. (*P* = 0.4688, df = 28, *n* = 15 biologically independent samples). Dots not displayed for the control group: 3.54, 2.52, and for the infected group: 2.51.

Secondary to microglia, astroglia may be recruited to the site of inflammation in the brain^77^. In order to analyze whether astrocytes contribute to the immune response in foci of *T. gondii* tachyzoites, we analyzed the adjacent section of the brain section, in which *T. gondii* tachyzoites were detected on gestation day 48, by immunohistochemistry for GFAP (Fig. 8a, b). Quantification of the GFAP labeling intensity in the tachyzoite related lesions and corresponding brain areas of the control group revealed no significant difference between the control and infection group (Fig. 8c). This indicates that parasitic lesions are not associated with focal or multifocal astroglioses in the brain of guinea pig fetuses 25 days after inoculation of dams with *T. gondii*.

## Discussion

This project provides novel and fundamental contributions to the understanding of the pathogenesis of congenital toxoplasmosis, specifically in the context of neocortex development and alterations of fetuses infected with *T. gondii*. We have previously established the guinea pig as a highly suitable animal model for human congenital toxoplasmosis in an attempt to overcome difficulties in performing clinical studies in pregnant women and in evaluating work in cell and tissue culture systems that fail to re-capitulate the complexity of a living animal^53^. Since all dams survived and no resorptions, abortions or stillbirth of fetuses were observed during the entire trial, our data confirm that oral administration of 100 *T. gondii* oocysts of the ME49 strain in the beginning of the second trimester of gestation, i.e., on gestation day 23, has no major impact on the guinea pig dam and offspring survival rate^53^. Moreover, we show that the administration of *T. gondii* oocysts to the dams has no negative effect on litter size.

We found no *T. gondii* DNA in the wide range of tissues and organs investigated, i.e., brain, heart, liver, lung, spleen, muscle, placenta and amniotic fluid, of fetuses dissected on gestation day 33, indicating that 10 days are not sufficient for a congenital transmission of the parasite and subsequent infection of the guinea pig fetus. When fetuses dissected on gestation days 40 and 48 were analyzed, *T. gondii* DNA was in two or more tissues or organs of each fetus, revealing that the parasite is stably transmitted to a larger extent to and replicates in the fetus 17 and 25 days after oral inoculation of the dam. These data are in line with a former study^52^, in which pregnant guinea pigs were orally inoculated with 100 tissue cysts of the 76 K strain. We previously observed that the duration a pregnant dam is infected affects the *T. gondii* load in the guinea pig offspring^53^. Indeed, this study shows that longer duration of *T. gondii* infection results in higher *T. gondii* DNA load in the extra-embryonic amniotic fluid. However, no differences in *T. gondii* DNA load were observed within the intra-embryonic tissues and organs. Given that in our previous study^53^, the duration of *T. gondii* incubation was up to 51 days, we suppose that the timespan, i.e., 8 days, between the two infection groups analyzed in this study was simply not long enough for any significant changes in the *T. gondii* DNA loads of the fetal tissues and organs to occur. Upon infection for 17 days, we observed no significant differences in the *T. gondii* DNA load between any of the fetal tissues and organs analyzed, which is consistent with previously published data^53^. Interestingly, when animals were analyzed after 25 days of inoculation, highest median *T. gondii* DNA loads in the fetus were observed in the brain. Moreover, the fetal brain possesses a remarkably high *T. gondii* positivity rate in both infection groups analyzed. Together, this confirms that the brain constitutes a preferred host organ of *T. gondii* in mammals including human^52,53,78–82^.

All *T. gondii* tachyzoites were located in the forebrain of guinea pig fetuses, mostly in the telencephalic basal nuclei and neocortex, which is in line with published data obtained in postnatal mammals including mice and human showing high *T. gondii* cyst burden to be present in the telencephalon including the amygdala, hippocampus, and various parts of the pallium such as the entorhinal, somatosensory, motor, orbital, frontal association, and visual cortex^83–85^. Moreover, we found *T. gondii* tachyzoites to be present in the choroid plexus and the lateral ventricle, thus providing further evidence for the notion that translocation across the choroid plexus and cerebrospinal fluid is a means for *T. gondii* to enter the brain^86,87^.

This study provides important new data on the in vivo host cells of *T. gondii* in the offspring neocortex before birth. Similar to in vivo data in the adult brain demonstrating that neurons and astrocytes represent targets of *T. gondii*, we observed *T. gondii* to infect neurons and astrocytes in the brain of guinea pig fetuses following the parasite’s vertical transmission^10,24,88,89^. In this regard it is of great importance that NPCs are almost exclusively present in high abundance in the prenatal neocortex^29–34,66^. Indeed, this study reveals that *T. gondii* infects NPCs in the brain of guinea pig fetuses congenitally infected with the parasite. These data are consistent with in vitro studies using a primary murine NPC culture^90,91^. As a result of congenital infection, we found the total number of BPs, which are known to generate the majority of cortical neurons^38–42^, to be markedly reduced in the neocortex of guinea pig fetuses during mid-neurogenesis. Specifically, we found the reduction in BP number to be mainly attributed to the Pax6+/Tbr2+ BP subtype population. On the assumption that sustained Pax6 expression in NPCs is linked to higher cell proliferation^42,92^, our data suggest that proliferative BPs committed to the neuronal lineage are most affected by *T. gondii* infection. As delaying the switch of symmetric proliferative NPC divisions to asymmetric neurogenic NPC divisions results in an exponential accumulation of neurons over time^93^, one would assume that the impact of reduced BP proliferation on neuron count manifests during final stages of neurogenesis. Indeed, our data reveal that the number of neurons is significantly reduced in the neocortex of fetuses infected with *T. gondii* on gestation 48, i.e., at end-neurogenesis when compared to healthy controls. These data confirm previous studies in children showing prenatal infection with *T. gondii* is associated with microcephaly^6,20^. Importantly, given that the reduction in neuron number is not primarily linked to an increase in neuronal cell death, our findings also indicate that, instead of neuron loss, impaired neurogenesis may underlie the reduction in neuron number observed in the neocortex of offspring congenitally infected with *T. gondii*.

Again, the reduction in BP cell counts observed in the neocortex of *T. gondii* infected fetuses was not associated with an increase in their cell death. Similarly, in vitro data using a primary murine NPC culture showed that an infection by the ME49 strain of *T. gondii* results in reduced NPC proliferation with no apoptosis induction^91^. In this regard it is interesting to note that *T. gondii* infection has been shown to result in dysregulation of the host cell cycle, specifically to arrest host cell cycle before progression to mitosis^22,25,94^. Moreover, recent data indicate that *T. gondii* induces a M cell cycle arrest by propagating chromosome segregation errors, mitotic spindle alteration and blockage of cytokinesis progression in primary bovine endothelial cells^27^. Therefore, one possible scenario might be that alterations in the BP cell cycle progression, specifically a blockage of its cell division rate, may explain the decrease in neurogenesis of the neocortex of *T. gondii* infected fetuses. Interestingly, *T. gondii* is able to inject effector proteins, e.g., rhoptry proteins, into cells it does not invade with the injected cells exceeding the infected cells many times over^95^. In this regard it is interesting to note, that *T. gondii* rhoptry protein ROP16 has been found to partially mediate cell cycle arrest in a human neuroblastoma cell line^96^. Hence, it could be assumed that *T. gondii* invasion and secretion of *T. gondii* effector proteins into distinct NPCs collectively subvert their functions leading to a global reduction in neurogenesis in infected fetuses. As an additional explanation, indirect environmental effects from the host response, e.g., from microglia may contribute to the observed reduction of the BP cell pool. Future studies will focus on the cellular and molecular alterations including the mode and rate of cell division of NPCs in the fetal brain induced by *T. gondii* as well as the specific molecular mechanisms that play a role in parasite invasion, proliferation, clearing of cells and injection of effector proteins in the fetal brain. To precisely determine *T. gondii* cell tropism in the fetal brain, highly sensitive methods, e.g., flow cytometry, should be used to complement the data provided by this study.

Moreover, our data indicate that congenital *T. gondii* infection does not affect the number of astrocytes in the fetal guinea pig brain, thus confirming the results of a recent in vitro study using a primary murine NPC culture, in which no alterations in the number of astrocytes were observed upon *T. gondii* infection^90^. NPCs are known to generate glial cells including astrocytes at later stages of cortical development once neurogenesis is mostly complete^97,98^. As we found no alteration in the number of Pax6+/Tbr2− NPC BPs, which are not committed to the neuronal lineage, it is very likely that the generation of astrocytes—contrary to that of neurons—by NCPs is not affected in the neocortex of guinea pig fetuses upon vertical *T. gondii* transmission, which in turn implies that distinct NPC subtypes differ in their susceptibility to *T. gondii* infection. However, as astrocytes—in contrast to neurons—maintain the ability to self-renew^99,100^, it cannot be ruled out that a decrease in astrocyte generation by NPCs was compensated by increased in astrocyte proliferation, leading to recovery of their quantity.

After birth, *T. gondii* infection of the central nervous system triggers an immune response by microglia, the resident immune cells of the brain and spinal cord, during the acute phase of infection^13,101^. We previously observed focal or multifocal microglioses in the neocortex of newborn guinea pigs prenatally infected with *T. gondii*^53^. During prenatal development, microglia start to colonize the brain concurrently with neurogenesis and prior to the generation of other glial cells^102–105^. As such, they might play various roles and are involved in neurogenesis, gliogenesis, and neuronal circuit formation^106^. Here, we show that the fetal guinea pig neocortex is populated by microglia as early as day 40 of gestation, i.e., during mid-neurogenesis. We found that congenital *T. gondii* infection results in the formation of activated microglia nodules in the guinea pig fetal brain in the second and third trimester of gestation, i.e., on gestation days 40 and 48, which suggests that microglia generate immune responses, specifically contributing to host defense of the brain against *T. gondii*, long before birth. Microglia are able to induce the activation of astrocytes, which may act as immunocompetent cells by secreting inflammatory cytokines and producing reactive oxygen species^77,107^. Indeed, previous data showed activated astrocytes to be associated with foci of microglia containing *T. gondii* tachyzoite clusters in the adult murine cortex following reactivation of chronic brain infection^108^. However, in this study no signs of focal astrogliosis associated with *T. gondii* tachyzoite clusters in the guinea pig brain were observed on gestation day 48. Given the time span of 25 days between inoculation of dams and analysis of the fetal brain, it is likely that this time window is too short for astrocytes to become activated to a large extent. Therefore, future studies using longer incubation periods and specific markers of astrocyte activation will reveal whether astrocytes - in addition to microglia - are part of the host defense against *T. gondii* in the fetal brain following congenital infection.

In summary, our data reveal that the neocortex is a major target region of *T. gondii* in fetuses of congenitally infected guinea pigs. We show for the first time that *T. gondii* infects NPCs, neurons and astrocytes in the fetal brain. Our results indicate that the reduction in neuron numbers observed in the neocortex of offspring congenitally infected with *T. gondii* is associated with its reduced generation and that microglia activation and recruitment to *T*. *gondii* tachyzoites is already established during the period of mid-neurogenesis. Together, our findings contribute considerably to the understanding of the cortical malformations observed in individuals congenitally infected with *T. gondii* and help to establish new treatments and solutions for congenital toxoplasmosis.

## Methods

### Ethics and animal welfare approval

All animal experiments were performed in accordance with German animal welfare legislation. The guinea pig study was approved by the Landesdirektion Sachsen (TVV 45/17, DD24.1-5131./390/47) and infection of cats to provide oocyst infection material was permitted by the ethics commission of the Animal Care and Use Committee of the German Lower Saxony State Office for Consumer Protection and Food Safety (reference number 33.19-42502-05-17A206). We have complied with all relevant ethical regulations for animal testing.

### Animals

Female Dunkin Hartley guinea pigs (*n* = 18, aged 3–6 months) were obtained from Charles River Laboratories (Ecully, France) and housed in the animal-care facility of the Institute of Parasitology, Faculty of Veterinary Medicine, University of Leipzig, Leipzig, Germany under previously described conditions^53^. In brief, dams were kept in groups of two in 50 × 70 × 50 cm wire mesh cages and provided water supplemented with 400 mg/l ascorbic acid, standard diet pellets (Altromin Spezialfutter, Lage, Germany) and fresh vegetables ad libitum. Room temperature was maintained at 19–25 °C and relative humidity at 55% (±10%). Light and dark cycle was set to 12:12 h. All dams were assessed daily based on a scoring system (Supplementary Table 1) during the entire trial.

### Mating and pregnancy examination

Dams were mated at the animal-care facility of the Institute of Parasitology, Faculty of Veterinary Medicine, University of Leipzig, Germany. Before, their sexual cycle was synchronized by oral administration of Altrenogest (Regumate® Equine 2,2 mg/ml, MSD Tiergesundheit, Unterschleißheim, Germany) at 0.22 mg/kg body weight once a day for 15 days^53^. Natural mating was initiated 2 days after the end of treatment by housing four females with one breeding ram for 4 days. Successful pregnancy was examined by ultrasound scan using GE Logiq 400 CL (pet mode, 7–10 MHz sample, GE Healthcare, Solingen, Germany). The day of successful mating is considered as gestation day 0 ± 2^53^. During ultrasound scan, the reproductive system, i.e., ovary and uterus wall, of all dams was examined.

### Serological investigation

Blood samples of all dams were collected from the Vena saphena lateralis immediately before infection and centrifuged (2500 × *g*, 10 min, 4 °C). Serum was analyzed by immunoblotting against *T. gondii* surface antigen p30 (SAG1) as previously described^109^, but using a peroxidase conjugated anti-guinea pig IgG (H + L, Jackson Immunoresearch Laboratories, West Grove, USA) instead of a peroxidase conjugated anti-mouse IgG.

### Infection

*T. gondii* oocysts (Strain ME49) were provided by the Institute of Parasitology, University of Veterinary Medicine Hannover, Germany. Oocysts were generated as previously described^53^. In brief, seronegative cats were given meat supplemented with brain, muscle, liver and spleen of guinea pigs chronically infected with *T. gondii*. Cat feces were purified using a sucrose solution gradient. Isolated oocysts were stored in 2% sulfuric acid at 4 °C for 1.5–2.5 months. Immediately before infection, 1 M sodium hydroxide solution was added to the oocyst suspension. Sporulated oocysts were quantified using a Neubauer-Improved cell counting chamber (Paul Marienfeld, Lauda-Königshofen, Germany). Oocysts were suspended in 500 µl phosphate buffered saline (PBS) and administered orally with a 16 G buttoned cannula (Henry Schein Dental, Langen, Germany). Guinea pigs were randomly divided into a control (*n* = 9) and infection (*n* = 9) group. Dams of the infection group were administrated 100 oocysts on gestation day 23. Dams of the control group received 500 µl PBS solution only.

### Euthanasia and dissection

Three dams and their corresponding litters of the control and infection group were euthanized by intraperitoneal injection of 500 mg/kg pentobarbital sodium on gestation days 33, 40 and 48, respectively. Fetuses were carefully dissected, and tissue samples were obtained from fetal brain, heart, liver, spleen, quadriceps femoris muscle, fetal placenta and amniotic fluid. Up to 600 mg of tissue was sampled per location. In case the entire tissue or organ weighed more, samples were taken randomly from at least 4 different regions. Except for the brain, the entire tissue and organ samples were frozen at −80 °C until further processing. Immediately after the removal of the brain, the cerebral hemispheres were separated. One cerebral hemisphere was frozen at −80 °C and used for qPCR. The other cerebral hemisphere was fixed in 4% paraformaldehyde for 4 days, stored in PBS at 4 °C and used for immunohistochemistry.

### Tissue homogenization and DNA extraction

Fetal tissue samples were processed using NucleoMag-Tissue kit (Macherey*-*Nagel, Düren, Germany) according to the manufacturer´s instructions with the following modifications: tissue samples (up to 600 mg) were incubated with lysis buffer (T1) supplied with the NucleoMag-Tissue kit (Macherey*-*Nagel, Düren, Germany) at ratio 1:2 and two stainless steel balls (diameter 6 mm, TIS Wälzkörpertechnologie, Gauting, Germany) were added. The sample solution was homogenized using a TissueLyser II (Qiagen, Hilden, Germany) at 3000 Hz for 60 s. Next, proteinase K of the NucleoMag-Tissue kit was added to the sample solution at ratio 1:10 and the solution was incubated at 56 °C overnight. 220 µl of lysate, corresponding to 100 mg of starting material, was used for all further extraction steps that were carried out using a King Fisher Flex resulting in 100 µl purified DNA (ThermoScientific, Waltham, USA).

### Quantitative PCR

A probe-based qPCR was applied, in which the 529 bp repeat element, characterized by high specificity and sensitivity for detection of *T. gondii*, was used as a sequence^110,111^. For all tissue and organ samples, qPCR was carried out as follows: primers and probes (MWG-Biotech, Ebersberg, Germany) were applied as previously described^112^ according to the assay protocol “Toxo529REP PCR” (Supplementary Table 3)^53^. An internal control according to^113^ was used for inhibition detection as previously described (Supplementary Table 3)^53,114^. When a negative result was due to PCR inhibition, the respective data was excluded from the data analysis. qPCR reactions were carried out in a CFX96 cycler (Biorad Laboratories, Kabelsketal, Germany) using 10 µl of purified DNA and the PerfeCTa MultiPlex qPCR ToughMix (VWR International, Dresden, Germany). qPCR conditions were as follows: 2 min at 50 °C and 10 min at 95 °C (initial denaturation). This was followed by 55 cycles, each consisting of 15 s at 95 °C (denaturation) and 1 min at 60 °C (hybridization and elongation). All qPCR results were evaluated using the CFX manager software version 1.6 (Biorad Laboratories, Kabelsketal, Germany). PCR results with a quantification cycle (Ct) equal to or higher than 40 were stated as Ct = 40 and considered as negative^53^. Brains of fetuses, whose Ct value was below 35 were included in the immunohistochemistry analysis. To establish a standard curve, tachyzoites were extracted from cell culture under the same conditions. Different negative extracts (guinea pig brain and muscle) were used for a 10-fold dilution series (2.8 × 10^1^–2.8 × 10^5^) in quintuplicates. The parameters for this semi-logarithmic standard curve are as follows: *y* = −3.406*x* + 36.36, *R*^2^ = 0.9977, *E* = 96.6%. Using this curve, Ct values were translated into genomic equivalents of tachyzoites.

### Immunocytochemistry

Cerebral hemispheres were processed and subjected to an immunohistochemistry protocol as described previously^115^. In brief, fixed hemispheres were dehydrated in 30% sucrose in PBS at room temperature, embedded in Tissue-Tek (Sakura Finetek, Staufen im Breisgau, Germany) and stored at −20 °C. Sections were cut at 30 μm using a Leica cryoystat (Leica CM1850, Leica Biosystems, Wetzlar, Germany) and stored at −20 °C. Complete brain was cut coronally.

For analysis of regional tropism of *T. gondii* tachyzoites, five randomly chosen sections per brain of all fetuses with Ct value below 35 were used. For analysis of *T. gondii* host cells, 6–8 sections per brain in close proximity to the section, in which *T. gondii* tachyzoites observed, were analyzed. For quantification of neurons, NPCs, apoptosis and astrocytes in the neocortex, the section at a medium position of the lateral ventricle with regard to the rostro-caudal axis per brain of all fetuses with Ct value below 35 was used. For analysis of focal micro- and astrogliosis, the adjacent section, to which *T. gondii* tachyzoites were observed, was evaluated.

Sections for Pax6, Tbr2 and Hu C/D immunohistochemistry were subjected to an antigen retrieval protocol by heating sections for 1 h at 90 °C in 0.01 M citrate buffer (pH 6). All sections were permeabilized with 0.3% Triton X-100 in PBS and quenched with 0.1 M glycine. Antibodies were diluted in 0.2% gelatine-PBS. Primary antibodies were incubated overnight at 4 °C and secondary antibodies were incubated for 1 h at room temperature. The following primary antibodies were used: Pax6 (1:200, rabbit, Biolegend, London, United Kingdom, 901301), Tbr2 (1:200, sheep, R&D Systems, Abingdon, United Kingdom, AF6166), Hu C/D (1:500, rabbit, Abcam, Amsterdam, Netherlands, ab184267), neurofilament H (1:500, chicken, Abcam, Amsterdam, Netherlands, ab8135), SAG1 (1:1200, mouse, Bio-Rad Laboratories, Feldkirchen, Germany, 9070-2020), MAP2 (1:1000, chicken, Abcam, Amsterdam, Netherlands, ab5392), GFAP (1:500, rabbit, antibodies.com, Cambridge, United Kingdom, A85419), caspase 3 (1:400, rabbit, Sigma-Aldrich Chemie, Steinheim, Germany, C8487) and IBA1 (1:500, rabbit, SynapticSystems, Göttingen, Germany, 234003). Pax6, Tbr2, Hu C/D, neurofilament H, MAP2 and GFAP antibodies have previously been successfully used in the immunohistochemistry analysis of fetal guinea pig brain tissue^66^. Dolichos biflorus agglutinin FITC-conjugated were obtained from Biozol (1:200, Eching, Germany, VEC-FL-1031- 5). Donkey secondary antibodies coupled to Alexa Fluor® 488 chicken (A11039), mouse (A21202), and rat (A21208), Alexa Fluor® 555 mouse (A31570) and rabbit (A31572), Alexa Fluor® 647 sheep (A21448) (1:500, Invitrogen, Darmstadt, Germany) and Alexa Fluor® 647 chicken (703-606-155) (1:500, Jackson ImmunoResearch Europe, Ely, UK) were used. All sections were counterstained with DAPI (1:500, Sigma, Taufkirchen, Germany), mounted in Mowiol (Merck Biosciences, Darmstadt, Germany), coverslipped and kept at 4 °C.

### Confocal laser scanning microscopy and deconvolution

Brain sections were imaged with a confocal laser scanning microscope (CLSM) Leica TCS SP8 (Leica Microsystems, Mannheim, Germany), using the software Leica Application Suite X (LAS-X 3.5.7). Images were acquired using the following microscope settings: bidirectional scan, scan speed 600 Hz, pinhole 1 Airy unit, and 3x line averaging. Sequential scanning of each channel separately was used to minimize cross talk between applied dyes. DAPI was excited at 405 nm (PMT detection range: 493–541 nm), Alexa Fluor® 488 at 488 nm (PMT detection range 493–541 nm), Alexa Fluor® 555 at 561 nm (HyD detection range 565–618 nm), and Alexa Fluor® 647 at 633 nm (HyD detection range 648–699 nm). The transmission (attenuator value) of each laser line was set so that the signal (8 bit-encoding) was just above saturation.

For analysis of regional tropism of *T. gondii* tachyzoites, tachyzoite clusters were manually determined under transmitted light using a ×40/1.10 W objective. For analysis of *T. gondii* host cells, a ×63/1.2 W objective was used and a scan resolution equivalent to a voxel size of 69 × 69 × 356 nm. Image stacks consisting of up to 61 layers were recorded to ensure that the entire parasite and compartment of the infected host cell was completely included. For quantification of neurons, NPCs and apoptosis, and of Iba1 and GFAP labeling intensity, a ×40/1.10 W objective was used and a scan resolution equivalent to a voxel size of 75 × 75 × 200 nm. Tile scans were recorded as z-stacks consisting of up to 5 layers.

Deconvolution of CLSM images, using Huygens Professional, version 20.10 (SVI, Hilversum, The Netherlands), was applied to reduce optical aberrations and to correct for the discrepancy in refractive indices (IR) of the embedding (IR = 1.49) and immersion media (IR = 1.33). Microscopic parameters were set according to the image metadata. A theoretical point spread function was used, and the following deconvolution parameters were set for batch processing with the Classic Maximum Likelihood Estimation (CMLE) algorithm: maximum iterations = 40, quality change threshold = 0.01, signal-to-noise ratio = 6.6667. Background for images of *T. gondii* host cell analysis and quantification of apoptosis and Iba1 and GFAP labeling intensity, all channels = 0. Background for images of quantification of neurons and NPCs: DAPI = 10.5, Alexa Fluor® 488 = 12, Alexa Fluor® 647 = 8.

### Data analysis

The ventricular zone (VZ), subventricular zone (SVZ), intermediate zone (IZ) and cortical plate (CP) were identified based on their cytoarchitecture and, in case of the SVZ, based on the presence and abundance of Tbr2 positive nuclei. In brief, the VZ represents a densely packed cell layer lining the lateral ventricle and whose nuclei exhibit radial morphology. The SVZ is located basally to the VZ and exhibits a looser and sparser cell arrangement than the VZ. The apical border of the SVZ is marked by the presence of a continuous Tbr2 positive nuclear layer.

The IZ contains very low cell density and lies between the SVZ and the CP. The CP is a cell layer adjacent to the IZ, which represents a high cell density and radially aligned nuclei.

Imaris 9.7.2 (Oxford Instruments, Abingdon, UK) was used for image analysis. For quantification of neurons, NPCs and apoptosis, and of Iba1 and GFAP labeling intensity, only the middle layer of the z-stacks was used.

For quantification of neurons and NPCs, colocalization between channels for DAPI and Hu C/D, DAPI and Pax6, DAPI and Tbr2, DAPI and Pax6 and Tbr2 was displayed. Intensity thresholds for corresponding channels were set as follows: DAPI = 10.5, Hu C/D = 9, Pax6 and Tbr2 = 13 each. The segmentation of colocalized signals was achieved by the following settings: surface detail = 0.2 μm, thresholding: background subtraction, diameters of largest sphere = 6 μm, manual threshold value: DAPI and Hu C/D = 2, DAPI and Pax6 = 8, DAPI and Tbr2 = 8, DAPI and Pax6 and Tbr2 = 4.5, seed point diameter = 6 μm, quality: DAPI and Hu C/D > 1, DAPI and Pax6 > 2, DAPI and Tbr2 > 2, DAPI and Pax6 and Tbr2 > 1.8. If nuclei of individual cells were displayed as merged objects by automatic segmentation, these objects were separated manually. The number of cells was quantified in a rectangle spanning the corresponding cortical zone with an apical edge length of 100 µm.

For astrocyte analysis in the neocortex, the number of voxels representing GFAP labeling with intensities greater than 50 out of 255 (8-bit coding) was quantified in a rectangle spanning entire cortical wall with an apical edge length of 100 µm. Apoptotic cells were quantified in a rectangle spanning the entire cortical wall with an apical edge length of 100 µm. Cells with a caspase-3 fluorescence intensity of more than 50 out of 255 (8-bit coding) and nuclear changes (e.g., segmentations) detected in the DAPI signal were considered as positive (Supplementary Fig. 4). For analysis of focal micro- and astrogliosis, Iba1 and GFAP labeling was analyzed in a 200 × 200 µm area around the tachyzoite clusters and of the same region in the brain of the control group was analyzed. The number of voxels representing Iba1 and GFAP labeling with intensities greater than 50 out of 255 (8-bit coding) was quantified and the results were expressed as voxels per µm^2^ (voxels projected onto an image area). Subsequently, microglia in this area were classified according to activated and non-activated using Imaris 9.9.1 (Oxford Instruments, Abingdon, UK). Surface rendering of all 5 *z*-stacks was used with the following parameters: surface detail = 0.2 μm, thresholding: absolute intensity 12.7, filter bounding box 250 x 250 nm. All objects consisting of less than 894 voxels (equivalent to a projected area of 1 µm^2^) were considered ramified, i.e., non-activated, and all objects that were larger were considered amoeboid, i.e., activated. Data were shown as a proportion of the respective microglia population. After data analysis, Adobe Photoshop was used for better visualization using selective gradation curve fitting of the weak DAPI-signal on the nuclei of *T. gondii* in Figs. 2 and 3 and Supplementary Fig. 1 and for a general gradation curve fitting of all channels in Figs. 4–7 and Supplementary Figs. 1 and 3.

### Statistics and reproducibility

A total of 18 guinea pig dams were obtained and randomly divided into a control (*n* = 9) and infection (*n* = 9) group. Each fetus was assessed as an individual sample. Numerical source data for all charts are provided as a Supplementary Data file. Statistical analysis was performed using Graphpad Prism 9.4.1 (GraphPad Software Inc., San Diego, USA) software. Normal distribution of all parameters was tested by Anderson-Darling test and Shapiro–Wilk test. *T. gondii* DNA loads obtained by qPCR analysis and number of apoptotic cells were not normally distributed and analyzed by Mann–Whitney test (for comparison of two groups) and Kruskal–Wallis test (for comparison of more than two groups) followed by Dunn’s post hoc test. All other data were normally distributed and analyzed using one-tailed unpaired *t* test. *P* values < 0.05 were considered statistically significant. Significance levels were split further as to **P < 0.01, ***P < 0.001, ****P < 0.0001.

### Reporting summary

Further information on research design is available in the Nature Portfolio Reporting Summary linked to this article.

### Supplementary information

Supplemental Information

Description of Additional Supplementary Files

Supplementary Data 1

Reporting Summary

## Data Availability

Data generated and analyzed during this study are included in this published article or can be obtained from the corresponding author (simone.fietz@vetmed.uni-leipzig.de) on request. Numerical source data for all plots and graphs in the manuscript can be found in Supplementary Data file.
