# Supplementary material for: Reduced neural progenitor cell count and cortical neurogenesis in guinea pigs congenitally infected with Toxoplasma gondii
Source: Commun Biol. 2023 Nov 27;6:1209. doi: 10.1038/s42003-023-05576-6 (PMC10682419; doi:10.1038/s42003-023-05576-6)
Supplement: Supplementary file 1 — Supplemental Information [file 42003_2023_5576_MOESM1_ESM.pdf]

SUPPLEMENTARY INFORMATION

Reduced neural progenitor cell count and cortical neurogenesis in guinea pigs congenitally infected with *Toxoplasma gondii*

Thomas Grochow[1,2], Britta Beck[1,2], Zaida Rentería-Solís,[2], Gereon Schares[3], Pavlo Maksimov[3,] Christina Strube[4], Lisa Raqué[5], Johannes Kacza[6], Arwid Daugschies[2] and Simone A. Fietz[1*]

* Corresponding author: simone.fietz@vetmed.leipzig.de

Supplementary Table 1: Scoring system used to evaluate the clinical condition of guinea pigs. If the additive score is greater than 3, the veterinarian and project manager will be informed, as well as the animals will be clinically examined in detail daily and additionally clinically observed at least once. In addition, appropriate measures are taken if necessary. If the additive score is greater than 5, painful conditions are assumed and 1.33 mg/ml metamizole sodium is added to the drinking water. If a score of 2 is achieved in the dehydration category, 60 ml/kg bw and a score of 3, 100 ml/kg bw of a 0.9% sodium chloride solution is applied subcutaneously. If an additive score of at least 2 is reached in the following categories: activity, hair coat, nutritional status, water and feed intake, then social stress conditions are assumed, and the animal is separated into an additional cage. Humane endpoint: an additive score of 10, assessment of two criteria with a score of 3, weight loss of 15% or more, ascites, abortion, inability to move, blindness, central nervous deficits or the presence of even individual clinical manifestations of corresponding severity (with or without connection to the scoring system presented or to the exposure of the animals to the actual animal experiment) the affected animals are euthanized. This is done according to the assessment of the responsible veterinarian.

**Criteria**	**0**	**1**	**2**	**3**
**Activity**	normal	isolated, abnormal posture	inactive or hyperactive	moribund
**Movement, gait**	normal	slightly uncoordinated or abnormal gait	uncoordinated or no longer fully applies limb or aversion to movement	swaying or does not sit up limb or paralysis
**Hair coat**	normal (smooth, shiny, close-fitting)	brittle	unkempt, wounds, loss of hair	bleeding or infected wounds, massive hair loss, automutilation
**Nutritional status**	normal (ribs palpable, abdomen barrel-shaped, no gluteal and axillary fat pads)	thin	loss of body fat	loss of muscle mass
**Water intake**	normal (50-100 ml/kg bw)	decreased or increased over 24 h	decreased or increased over 48 h	permanent drinking or no water intake over 24 h
**Food intake**	normal (30-50 g pellets/kg bw)	decreased or increased over 24 h	decreased or increased over 48 h	obese or inappetence over 48 h
**Urine**	normal (straw-yellow)		abnormal color / quantity	no urine output over 24 h or wet perineum
**Feces**	normal (dry, clumped)	mushy, moist	diarrhea or decreased fecal output	diarrhea over 24 h, bloody admixtures, no defecation over 24 h
**Breathing**	normal, 100-150 breaths/min	elevated, flat	elevated, abdominal	dyspnea, irregular, cyanotic colored mucous membranes
**Vocalization**	normal, minor with manipulation	none in case of manipulation	high in case of manipulation	
**Dehydration**	none	reduced skin elasticity	skin fold persists	skin fold remains, sunken eyes
**Eyes**	normal	dry, dull	eye discharge	lids stuck together, hemophthalmos, exophthalmos
**Nose**	normal	dried out	minor nasal discharge	high-grade discharge, stuck
**Mucosal color**	pale pink		reddened or pale	icteric, high-grade redness or cyanotic
**Vagina**	dry, no discharge	swollen	clear discharge	cloudy, purulent or bloody discharge

Supplementary Table 2: *T. gondii* qPCR results separated by tissue and organ for each dam and corresponding fetuses of the infection groups. Data on grey background represent Ct values for *T. gondii* in the heart, liver and spleen of dams inoculated with *T. gondii* on gestation day 23 or of control dams dissected on gestation 30, 40 or 48, respectively. Data on white background represent Ct values for *T. gondii* in the fetal brain, heart, liver, lung, spleen, muscle, placenta and amniotic fluid of corresponding dams inoculated with *T. gondii* on gestation day 23 or of control dams dissected on gestation 30, 40 or 48, respectively. A minus sign represents a negative result defined by a quantification cycle (Ct) equal to or higher than 40 in the qPCR analysis. No result could be obtained in the qPCR analysis for samples with the designation "IH", indicating qPCR inhibition. The term "ND" represents values that were not determined. Fetal positivity rate represents the percentage of positive samples for a specific tissue or organ from all fetuses dissected on the same gestation day. Given the high number of qPCR inhibitions in the fetal liver and lung samples, fetal positivity rate was not determined in these two organs.

Line	Dam no.	Fetus no.	Infection Dose	Day of gestation	Brain	Heart	Liver	Lung	Spleen	Muscle	Placenta	Amniotic fluid
1	1		100	33	ND	29.67	IH	ND	IH	ND	ND	ND
2	1	A	100	33	-	-	IH	-	-	IH	-	-
3	1	B	100	33	-	-	-	-	-	-	IH	-
4	1	C	100	33	-	-	IH	-	-	-	-	-
5	1	D	100	33	-	-	IH	-	-	-	-	-
6	2		100	33	ND	32.26	35.71	ND	IH	ND	ND	ND
7	2	A	100	33	-	-	IH	-	-	-	IH	-
8	2	B	100	33	-	-	IH	-	-	-	IH	-
9	2	C	100	33	-	-	-	-	-	-	IH	-
10	2	D	100	33	-	-	IH	-	-	-	-	-
11	3		100	33	ND	30.90	35.66	ND	IH	ND	ND	ND
12	3	A	100	33	-	-	-	-	-	-	-	-
13	3	B	100	33	-	-	-	-	-	-	-	-
14	4		100	40	ND	28.26	32.71	ND	IH	ND	ND	ND
15	4	A	100	40	31.30	31.25	IH	IH	31.41	32.43	27.87	39.92
16	4	B	100	40	31.77	31.54	IH	IH	31.77	-	29.66	-
17	5		100	40	ND	29.43	33.39	ND	IH	ND	ND	ND
18	5	A	100	40	33.27	31.19	IH	31.68	31.48	-	31.01	-
19	5	B	100	40	30.93	29.74	IH	IH	34.10	32.50	30.68	-
20	5	C	100	40	39.05	-	IH	37.39	-	-	29.53	-
21	5	D	100	40	33.33	37.02	IH	-	36.36	35.46	33.70	-
22	6		100	40	ND	30.78	34.18	ND	IH	ND	ND	ND
23	6	A	100	40	35.58	36.08	IH	-	32.66	37.21	30.83	-
24	6	B	100	40	-	32.56	IH	IH	-	-	33.73	-
25	6	C	100	40	36.96	-	IH	-	-	-	IH	38.76
26			**Fetal positivity rate**		**89%**	**78%**	**ND**	**ND**	**67%**	**44%**	**100%**	**22%**
27	7		100	48	ND	29.23	IH	ND	IH	ND	ND	ND
28	7	A	100	48	33.50	34.40	IH	IH	-	35.50	22.94	30.52
29	7	B	100	48	31.19	34.47	IH	IH	-	38.16	25.06	29.70
30	7	C	100	48	36.30	31.64	IH	IH	-	37.04	24.64	28.09
31	7	D	100	48	35.28	34.73	IH	IH	-	IH	31.24	27.58
32	8		100	48	ND	30.36	IH	ND	IH	ND	ND	ND
33	8	A	100	48	33.57	34.51	IH	IH	36.27	-	IH	25.78
34	8	B	100	48	33.31	IH	IH	IH	IH	IH	30.49	31.69
35	8	C	100	48	30.05	33.43	IH	IH	36.09	IH	27.66	33.59
36	9		100	48	ND	31.88	IH	ND	IH	ND	ND	ND
37	9	A	100	48	31.92	32.02	IH	35.63	-	35.00	31.89	31.99
38	9	B	100	48	29.72	IH	IH	IH	38.67	-	33.85	30.42
39	9	C	100	48	30.56	34.18	IH	IH	34.73	34.56	23.63	26.97
40	9	D	100	48	27.06	32.23	IH	IH	32.52	34.58	22.29	29.91
41	9	E	100	48	31.44	31.39	IH	IH	36.02	34.48	28.38	23.88
42			**Fetal positivity rate**		**100%**	**100%**	**ND**	**ND**	**58%**	**78%**	**100%**	**100%**

Line	Dam no.	Fetus no.	Infection Dose	Day of gestation	Brain	Heart	Liver	Lung	Spleen	Muscle	Placenta	Amniotic fluid
43	10	A	0	33	-	-	-	-	-	-	IH	-
44	10	B	0	33	-	-	IH	-	-	-	IH	-
45	10	C	0	33	-	-	IH	-	-	-	IH	-
46	10	D	0	33	-	-	IH	-	-	-	IH	-
47	11		0	33	ND	-	-	ND	IH	ND	ND	ND
48	11	A	0	33	-	-	IH	-	-	-	IH	-
49	11	B	0	33	-	-	IH	-	-	-	IH	-
50	11	C	0	33	-	-	-	-	-	-	IH	-
51	12		0	33	ND	IH	-	ND	IH	ND	ND	ND
52	12	A	0	33	-	-	IH	-	-	-	IH	-
53	12	B	0	33	-	-	-	-	-	-	IH	-
54			**Fetal positivity rate**		**0%**	**0%**	**ND**	**ND**	**0%**	**0%**	**ND**	**0%**
55	13		0	40	ND	-	-	ND	IH	ND	ND	ND
56	13	A	0	40	-	-	IH	IH	-	IH	IH	-
57	13	B	0	40	-	-	IH	IH	-	-	IH	-
58	14		0	40	ND	-	-	ND	IH	ND	ND	ND
59	14	A	0	40	-	-	IH	IH	-	IH	-	-
60	14	B	0	40	-	-	IH	-	-	-	IH	-
61	14	C	0	40	-	-	IH	IH	-	IH	IH	-
62	15		0	40	ND	IH	-	ND	IH	ND	ND	ND
63	44	A	0	40	-	-	IH	IH	-	IH	IH	-
64	44	B	0	40	-	-	IH	IH	-	IH	-	-
65			**Fetal positivity rate**		**0%**	**0%**	**ND**	**ND**	**0%**	**0%**	**ND**	**0%**
66	16		0	48	ND	-	-	ND	IH	ND	ND	ND
67	16	A	0	48	-	IH	IH	IH	-	-	IH	-
68	17		0	48	ND	-	-	ND	IH	ND	ND	ND
69	17	A	0	48	-	-	IH	IH	-	-	IH	-
70	17	B	0	48	-	-	IH	-	IH	-	-	-
71	18		0	48	ND	-	-	ND	IH	ND	ND	ND
72	18	A	0	48	-	-	IH	IH	IH	IH	IH	-
73	18	B	0	48	-	-	IH	IH	IH	IH	IH	-
74			**Fetal positivity rate**		**0%**	**0%**	**ND**	**ND**	**0%**	**0%**	**0%**	**0%**

Supplementary Table 3: Primers, probes and their final concentrations in *Toxoplasma gondii* real-time qPCR assay.

Assay	Names of primers and probes	Sequences of primers and probes 5′–3′	Probe labelling	Final concentration	Reference
Toxo529REP PCR	TalF	TGG TTG GGA AGC GAC GAG AG		800 nM	Talabani et al. 2009
	TalR	CAT CAC CAC GAG GAA AGC GTC		800 nM	Talabani et al. 2009
	TalP1_FAM	TGT CGT GCC AGC TGC ATT A	5′-FAM, 3′-BHQ1	200 nM	Talabani et al. 2009
Internal control PCR, IC2 PCR	EGFP1-F	GAC CAC TAC CAG CAG AAC AC		500 nM	Hoffmann et al. 2006
	EGFP2-R	GAA CTC CAG CAG GAC CAT G		500 nM	Hoffmann et al. 2006
	EGFP1-Hex	AGC ACC CAG TCC GCC CTG AGC A	5′-HEX, 3′-BHQ1	160 nM	Hoffmann et al. 2006

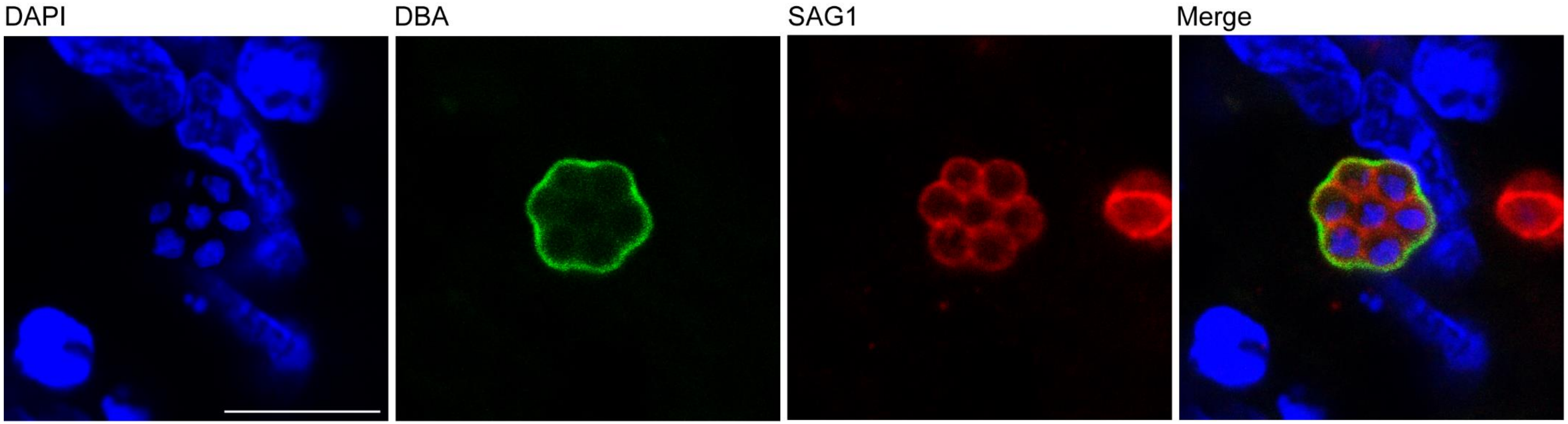

Supplementary Fig. 1: Identification of *T. gondii* tachyzoites in tissue cysts. DAPI staining (blue), DBA staining (green) and immunofluorescence for SAG1 (red) on a 30 µm brain cryosection of a guinea pig fetus of a dam inoculated with *T. gondii* on gestation day 23 and dissected on day 48. Scale bar, 10 µm.

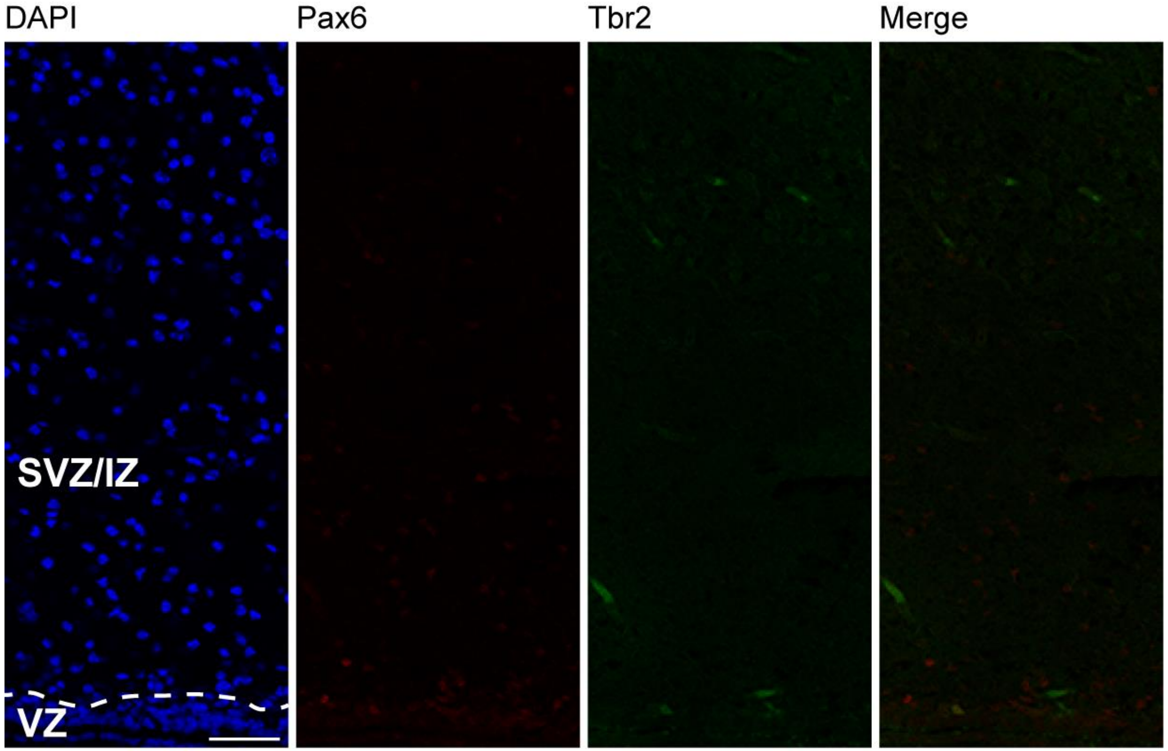

Supplementary Fig. 2: Absence of Pax6 and Tbr2 expression in the fetal guinea pig neocortex on gestation day 48. DAPI staining (blue) and immunofluorescence labeling for Pax6 (red) and Tbr2 (green) on 30 µm brain cryosections of a guinea pig fetus dissected on day 48. Scale bar, 50 µm. The dashed line indicates the border between VZ and SVZ/IZ.

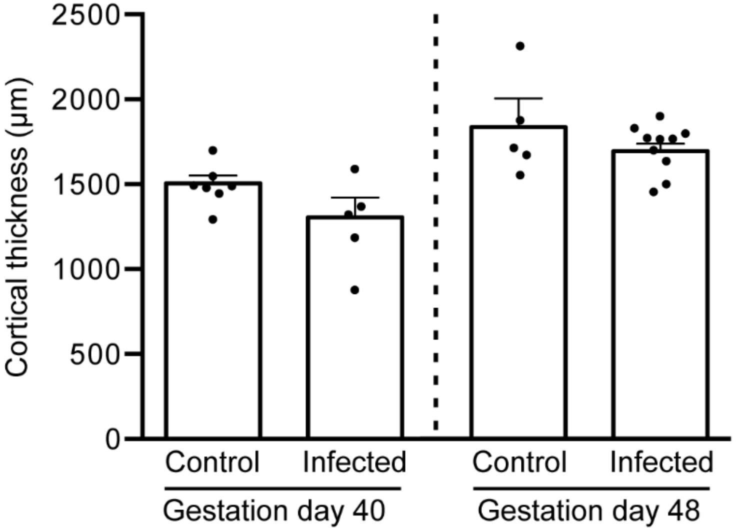

Supplementary Fig. 3: Cortical thickness of the fetal guinea pig neocortex. Center line, mean; error bar, SEM. Data of the infection groups are from all guinea pig brains with Ct value below 35 as determined by qPCR for *T. gondii* (*n* = 5 biologically independent samples for gestation day 40, *n* = 10 biologically independent samples, for gestation day 48, Supplementary Table 2). Data of the control group are as follows: *n* = 7 biologically independent samples for gestation day 40, *n* = 5 biologically independent samples for gestation day 48.

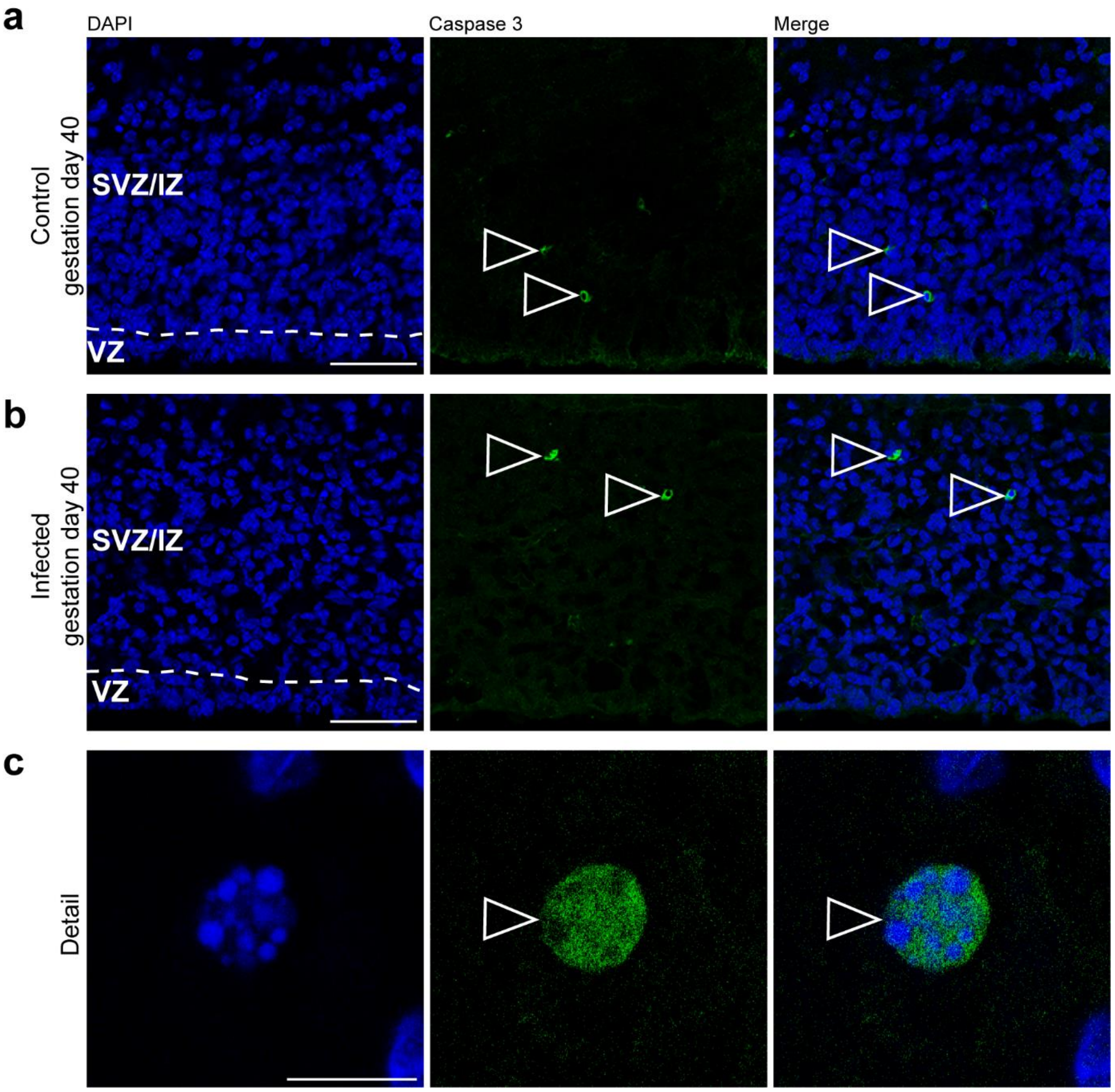

Supplementary Fig. 4: Identification of apoptotic cells by immunohistochemistry for activated caspase 3. DAPI staining (blue) and immunohistochemistry for activated caspase 3 (green) on a 30 µm brain cryosection of a guinea pig fetus of control (a) or infected (b) dam dissected on gestation day 40. Arrowheads indicate activated caspase 3-expressing cells. Scale bars, 50 (**a, b**) , 10 µm (**c**). VZ, ventricular zone; SVZ, subventricular zone; IZ, intermediate zone.

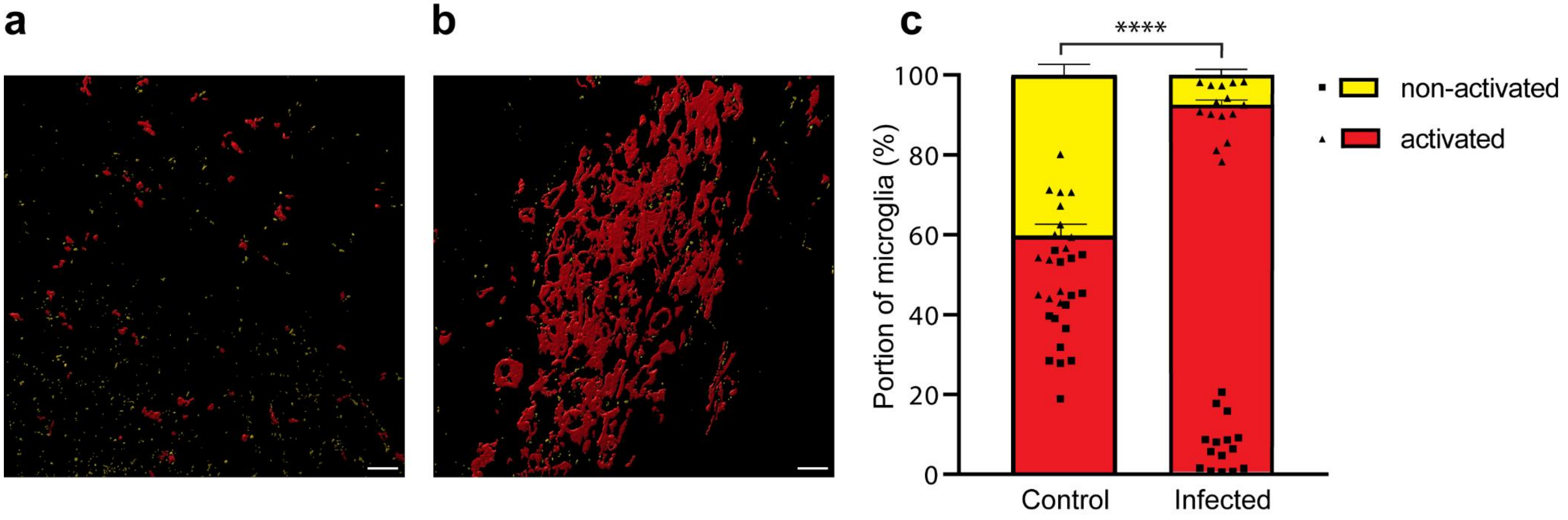

Supplementary Fig. 5: Microglia classification. Immunohistochemistry for activated (red) and non-activated (red) microglia on 30 µm brain cryosection of a fetus of a control (**a**) or infected (**b**) dam dissected on gestation day 40. Scale bars, 15 µm. Imaris surface classification was used to analyze the microglia accordingly. (**c**) Classification of the proportions of voxels representing activated or non-activated microglia. Microglia were analyzed in a 200 x 200 µm area including a tachyzoite cluster of guinea pig fetuses of dams inoculated with *T. gondii* on gestation day 23 and from a 200 x 200 µm area covering the same brain region of control fetuses, dissected on gestation day 40 and 48. Center line, mean; error bar, SEM. Data are from 15 *T. gondii* tachyzoite clusters observed in 5 fetuses dissected on gestation day 40 and 48 (Figure 2) and the corresponding regions of the control groups. ****, $P < 0.0001$, df = 28.
