# Supplementary material for: Reduced neural progenitor cell count and cortical neurogenesis in guinea pigs congenitally infected with Toxoplasma gondii
Source: Commun Biol. 2023 Nov 27;6:1209. doi: 10.1038/s42003-023-05576-6 (PMC10682419; doi:10.1038/s42003-023-05576-6)
Supplement: Supplementary file 2 — Description of Additional Supplementary Files [file 42003_2023_5576_MOESM2_ESM.docx]

Description of Additional Supplementary Files

**File name:** Supplementary Data 1

**Description:** Numerical source data for the graphs shown in this study.
