# Supplementary material for: Reduced neural progenitor cell count and cortical neurogenesis in guinea pigs congenitally infected with Toxoplasma gondii
Source: Commun Biol. 2023 Nov 27;6:1209. doi: 10.1038/s42003-023-05576-6 (PMC10682419; doi:10.1038/s42003-023-05576-6)
Supplement: Supplementary file 4 — Reporting Summary [file 42003_2023_5576_MOESM4_ESM.pdf]

nature portfolio

Corresponding author(s): Simone Fietz
Last updated by author(s): 20/10/23

Reporting Summary

Nature Portfolio wishes to improve the reproducibility of the work that we publish. This form provides structure for consistency and transparency in reporting. For further information on Nature Portfolio policies, see our Editorial Policies and the Editorial Policy Checklist.

Statistics

For all statistical analyses, confirm that the following items are present in the figure legend, table legend, main text, or Methods section.

n/a	Confirmed	
☐	☒	The exact sample size (*n*) for each experimental group/condition, given as a discrete number and unit of measurement
☐	☒	A statement on whether measurements were taken from distinct samples or whether the same sample was measured repeatedly
☐	☒	The statistical test(s) used AND whether they are one- or two-sided<br>*Only common tests should be described solely by name; describe more complex techniques in the Methods section.*
☐	☒	A description of all covariates tested
☐	☒	A description of any assumptions or corrections, such as tests of normality and adjustment for multiple comparisons
☐	☒	A full description of the statistical parameters including central tendency (e.g. means) or other basic estimates (e.g. regression coefficient) AND variation (e.g. standard deviation) or associated estimates of uncertainty (e.g. confidence intervals)
☐	☒	For null hypothesis testing, the test statistic (e.g. *F*, *t*, *r*) with confidence intervals, effect sizes, degrees of freedom and *P* value noted<br>*Give P values as exact values whenever suitable.*
☒	☐	For Bayesian analysis, information on the choice of priors and Markov chain Monte Carlo settings
☒	☐	For hierarchical and complex designs, identification of the appropriate level for tests and full reporting of outcomes
☒	☐	Estimates of effect sizes (e.g. Cohen's *d*, Pearson's *r*), indicating how they were calculated

Our web collection on statistics for biologists contains articles on many of the points above.

Software and code

Policy information about availability of computer code

Data collection	*Provide a description of all commercial, open source and custom code used to collect the data in this study, specifying the version used OR state that no software was used.*
Data analysis	*Provide a description of all commercial, open source and custom code used to analyse the data in this study, specifying the version used OR state that no software was used.*

For manuscripts utilizing custom algorithms or software that are central to the research but not yet described in published literature, software must be made available to editors and reviewers. We strongly encourage code deposition in a community repository (e.g. GitHub). See the Nature Portfolio guidelines for submitting code & software for further information.

Data

Policy information about availability of data

All manuscripts must include a data availability statement. This statement should provide the following information, where applicable:

- Accession codes, unique identifiers, or web links for publicly available datasets
- A description of any restrictions on data availability
- For clinical datasets or third party data, please ensure that the statement adheres to our policy

Data generated and analyzed during this study are included in this published article or can be obtained from the corresponding author on request.

Research involving human participants, their data, or biological material

Policy information about studies with human participants or human data. See also policy information about sex, gender (identity/presentation), and sexual orientation and race, ethnicity and racism.

Reporting on sex and gender	*Use the terms sex (biological attribute) and gender (shaped by social and cultural circumstances) carefully in order to avoid confusing both terms. Indicate if findings apply to only one sex or gender; describe whether sex and gender were considered in study design; whether sex and/or gender was determined based on self-reporting or assigned and methods used. Provide in the source data disaggregated sex and gender data, where this information has been collected, and if consent has been obtained for sharing of individual-level data; provide overall numbers in this Reporting Summary. Please state if this information has not been collected. Report sex- and gender-based analyses where performed, justify reasons for lack of sex- and gender-based analysis.*
Reporting on race, ethnicity, or other socially relevant groupings	*Please specify the socially constructed or socially relevant categorization variable(s) used in your manuscript and explain why they were used. Please note that such variables should not be used as proxies for other socially constructed/relevant variables (for example, race or ethnicity should not be used as a proxy for socioeconomic status). Provide clear definitions of the relevant terms used, how they were provided (by the participants/respondents, the researchers, or third parties), and the method(s) used to classify people into the different categories (e.g. self-report, census or administrative data, social media data, etc.) Please provide details about how you controlled for confounding variables in your analyses.*
Population characteristics	*Describe the covariate-relevant population characteristics of the human research participants (e.g. age, genotypic information, past and current diagnosis and treatment categories). If you filled out the behavioural & social sciences study design questions and have nothing to add here, write "See above."*
Recruitment	*Describe how participants were recruited. Outline any potential self-selection bias or other biases that may be present and how these are likely to impact results.*
Ethics oversight	*Identify the organization(s) that approved the study protocol.*

Note that full information on the approval of the study protocol must also be provided in the manuscript.

Field-specific reporting

Please select the one below that is the best fit for your research. If you are not sure, read the appropriate sections before making your selection.

☒ Life sciences ☐ Behavioural & social sciences ☐ Ecological, evolutionary & environmental sciences

For a reference copy of the document with all sections, see nature.com/documents/nr-reporting-summary-flat.pdf

Life sciences study design

All studies must disclose on these points even when the disclosure is negative.

Sample size	For a pilot study, a group size of 12 animals is recommended (DOI: 10.1002/pst.185). In our previous study (DOI: 10.1186/s13071-021-04890-4), 3 dams were used per group because each dam has approximately 4 offspring per litter. In the previous study, sufficient data could be obtained with this group size, so we kept this group size for the current study.
Data exclusions	No data were excluded.
Replication	3 dams per group were used under the same conditions.
Randomization	All animals were obtained at the same age at the same time. Immediately after arrival, animals were assigned to the infection and control groups. Due to the fact that the animals were not visually distinguishable, the assignment was random.
Blinding	Grooming and mating of the animals was performed under blinded conditions. Data analysis was performed unblinded by the same rater.

Reporting for specific materials, systems and methods

We require information from authors about some types of materials, experimental systems and methods used in many studies. Here, indicate whether each material, system or method listed is relevant to your study. If you are not sure if a list item applies to your research, read the appropriate section before selecting a response.

Materials & experimental systems

n/a	Involved in the study
☐	☒ Antibodies
☒	☐ Eukaryotic cell lines
☒	☐ Palaeontology and archaeology
☐	☒ Animals and other organisms
☒	☐ Clinical data
☒	☐ Dual use research of concern
☒	☐ Plants

Methods

n/a	Involved in the study
☒	☐ ChIP-seq
☒	☐ Flow cytometry
☒	☐ MRI-based neuroimaging

Antibodies

Antibodies used

Pax6 (1:200, rabbit, Biolegend, London, United Kingdom, 901301), Tbr2 (1:200, sheep, R&D Systems, Abingdon, United Kingdom, AF6166), Hu C/D (1:500, rabbit, Abcam, Amsterdam, Netherlands, ab184267), neurofilament H (1:500, chicken, Abcam, Amsterdam, Netherlands, ab8135), SAG1 (1:1200, mouse, Bio-Rad Laboratories, Feldkirchen, Germany, 9070-2020), MAP2 (1:1000, chicken, Abcam, Amsterdam, Netherlands, ab5392), GFAP (1:500, rabbit, antibodies.com, Cambridge, United Kingdom, A85419), caspase 3 (1:400, rabbit, Sigma-Aldrich Chemie, Steinheim, Germany, C8487) and IBA1 (1:500, rabbit, SynapticSystems,, Göttingen, Germany, 234003).

Validation

All antibodies used in the study have been successfully applied in immunohistochemistry on brain tissue sections of various species including other rodents such as mouse, rat and rabbit as well as human, marmoset, macaque, ferret, tree shrew and/or wallaby:

Betizeau, M. et al. Precursor diversity and complexity of lineage relationships in the outer subventricular zone of the primate. Neuron. 80, 442–457 (2013).
Chen, W. et al. Patchouli alcohol improved diarrhea-predominant irritable bowel syndrome by regulating excitatory neurotransmission in the myenteric plexus of rats. Frontiers in pharmacology 13, 943119 (2022).
Fietz, S. A., Namba, T., Kirsten, H., Huttner, W. B. & Lachmann, R. Signs of Reduced Basal Progenitor Levels and Cortical Neurogenesis in Human Fetuses with Open Spina Bifida at 11-15 Weeks of Gestation. J Neurosci. 40, 1766–1777 (2020).
Gibson, J. M. et al. Perinatal methadone exposure attenuates myelination and induces oligodendrocyte apoptosis in neonatal rat brain. Exp Biol Med. 247, 1067–1079 (2022).
Hwang, S.-H., Somatilaka, B. N., White, K. & Mukhopadhyay, S. Ciliary and extraciliary Gpr161 pools repress hedgehog signaling in a tissue-specific manner. eLife 10 (2021).
Jin, L. et al. Astrocytic SARM1 promotes neuroinflammation and axonal demyelination in experimental autoimmune encephalomyelitis through inhibiting GDNF signaling. Cell Death & Disease 13, 759 (2022).
Kalusa, M., Heinrich, M. D., Sauerland, C., Morawski, M. & Fietz, S. A. Developmental differences in neocortex neurogenesis and maturation between the altricial dwarf rabbit and precocial guinea pig. Front Neuroanat. 15, 678385 (2021).
Kelava, I. et al. Abundant occurrence of basal radial glia in the subventricular zone of embryonic neocortex of a lissencephalic primate, the common marmoset Callithrix jacchus. Cereb Cortex. 22, 469–481 (2012).
Martens, Y. A. et al. Generation and validation of APOE knockout human iPSC-derived cerebral organoids. STAR Protocols 2, 100571 (2021).
Römer, S. et al. Neural Progenitors in the Developing Neocortex of the Northern Tree Shrew (Tupaia belangeri) Show a Closer Relationship to Gyrencephalic Primates Than to Lissencephalic Rodents. Front Neuroanat. 12, 29 (2018).
Sauerland, C. et al. The basal radial glia occurs in marsupials and underlies the evolution of an expanded neocortex in therian mammals. Cereb Cortex. 28, 145–157 (2018).
Yin, G., Lin, Y., Wang, P., Zhou, J. & Lin, H. Upregulated lncARAT in Schwann cells promotes axonal regeneration by recruiting and activating proregenerative macrophages. Molecular medicine (Cambridge, Mass.) 28, 76 (2022).
Zeng, Y. et al. The Impact of Particulate Matter (PM2.5) on Human Retinal Development in hESC-Derived Retinal Organoids. Front Cell Dev Biol. 9, 607341 (2021).

Specifically, the antibodies used for the detection of Pax6, Tbr2, Hu C/D, neurofilament H, MAP2 and GFAP in our study have previously been successfully used in the immunohistochemistry analysis of fetal guinea pig brain tissue (Kalusa et al., 2021). For all antibodies, specific information regarding their dilution, source, manufacturer, catalog number are provided in the Methods section of manuscript. Details regarding the species reactivity are provided by the manufacturer website and are as follows:

Pax6, verified reactivity: human, mouse, rat
Tbr2, verified reactivity: human (BLAST alignment revealed 97% identity between the human and guinea pig Tbr2 immunogen)
Hu C/D, verified reactivity: mouse, rat, human
Neurofilament H, verified reactivity: mouse, rat
MAP2, verified reactivity: mouse, rat
GFAP, verified reactivity: human, horse, bovine, porcine, rat, mouse
caspase 3, verified reactivity: rat, bovine, human, pig, canine, mouse.

Animals and other research organisms

Policy information about studies involving animals; ARRIVE guidelines recommended for reporting animal research, and Sex and Gender in Research

Laboratory animals	Dunkin Hartley female guinea pig, Charles River Laboratories (Ecully, France) .
Wild animals	*Provide details on animals observed in or captured in the field; report species and age where possible. Describe how animals were caught and transported and what happened to captive animals after the study (if killed, explain why and describe method; if released, say where and when) OR state that the study did not involve wild animals.*
Reporting on sex	All animals were female.
Field-collected samples	*For laboratory work with field-collected samples, describe all relevant parameters such as housing, maintenance, temperature, photoperiod and end-of-experiment protocol OR state that the study did not involve samples collected from the field.*
Ethics oversight	All animal experiments were performed in accordance with German animal welfare legislation. The guinea pig study was approved by the Landesdirektion Sachsen (TVV 45/17, DD24.1-5131./390/47) and infection of cats to provide oocyst infection material was permitted by the ethics commission of the Animal Care and Use Committee of the German Lower Saxony State Office for Consumer Protection and Food Safety (reference number 33.19 42502-05-17A206).

Note that full information on the approval of the study protocol must also be provided in the manuscript.
